# Causal role of blood metabolites in HER-positive and HER-negative breast cancer: a Mendelian randomization (MR) study

**DOI:** 10.18632/aging.206042

**Published:** 2024-08-02

**Authors:** Jian Yue, Huiying Fang, Sheng Chen, Lei Gu, Guosheng Ren

**Affiliations:** 1 Chongqing Key Laboratory of Molecular Oncology and Epigenetics, The First Affiliated Hospital of Chongqing Medical University, Chongqing, China; 2 Department of Breast and Thyroid Surgery, The First Affiliated Hospital of Chongqing Medical University, Chongqing, China; 3 Department of Breast Surgery, Gaozhou People’s Hospital, Gaozhou, China; 4 Department of Breast Cancer Center, Chongqing Key Laboratory for Intelligent Oncology in Breast Cancer (iCQBC), Chongqing University Cancer Hospital, Chongqing, China; 5 Department of Pulmonary and Critical Care Medicine, The First Affiliated Hospital of Soochow University, Suzhou, China

**Keywords:** blood metabolites, breast cancer, HER, Mendelian randomization

## Abstract

Background: Previous studies provide evidence that *in vivo* metabolites are associated with breast cancer (BC). However, the causal relationship between blood metabolites and BC remains unclear.

Method: Comprehensive two-sample Mendelian randomization analysis was conducted to determine the causal association between 1400 publicly available genetic data on metabolic factors and human epidermal growth factor receptor positive (HER+) BC or HER- BC in this study.

Result: Epiandrosterone sulfate levels (OR = 1.07, 95% CI = 1.02 ~ 1.10, *p* = 0.0013), 5alpha-androstan-3beta,17beta-diol monosulfate (2) levels (OR = 1.07, 95% CI = 1.03 ~ 1.12, *p* = 0.0012), glycohyocholate levels (OR = 0.85, 95% CI = 0.77 ~ 0.93, *p* = 0.0007) and etiocholanolone glucuronide levels (OR = 1.12, 95% CI = 1.05 ~ 1.20, *p* = 0.0013) were causally correlated with HER+ BC. 5 metabolites were causally correlated with HER- BC: Vanillic acid glycine levels (OR = 1.14, 95% CI = 1.06 ~ 1.22, *p* = 0.0003), Thyroxine levels (OR = 1.26, 95% CI = 1.11 ~ 1.44, *p* = 0.0004), 1-palmitoyl-2-linoleoyl-GPI (16:0/18:2) levels (OR = 0.86, 95% CI = 0.79 ~ 0.94, *p* = 0.0010), N-acetylphenylalanine levels (OR = 1.12, 95% CI = 1.05 ~ 1.19, *p* = 0.0007) and Glucose-to-mannose ratio (OR = 1.15, 95% CI = 1.06 ~ 1.24, *p* = 0.0008). Two common causally related metabolites were identified: Gamma-glutamyl glutamate and X-12849 levels.

Conclusions: Our study has respectively demonstrated the connection between blood metabolites and HER+ or HER- BC by genetic means, thereby offering opportunities for therapeutic targets.

## INTRODUCTION

The most recent report from CA (A Cancer Journal for Clinicians) indicates that breast cancer (BC) is currently the most commonly diagnosed cancer among women, accounting for 31% of female cancer cases in 2023. It remains the second leading cause of cancer-related mortality in women, accounting for 15% of such instances [1]. The ErbB family comprises receptor tyrosine kinases, including human epidermal growth factor receptors (HER) 1/2/3/4, situated on the cellular membrane and responsive to a diverse range of ligands [2]. These receptors, capable of homo- or heterodimerization, play a crucial role in normal cell development but can lead to cancer through dysregulation, driving abnormal cell growth and survival through intricate signaling pathways [3, 4]. Among these receptors, HER2 has been extensively studied and is a primary target for treatment. Overexpression of HER2 is observed in approximately 15–20% of breast cancer cases and is associated with a poorer prognosis [2, 5]. Notably, HER3 lacks intrinsic kinase activity but can form heterodimers with HER2 (and/or HER1), significantly enhancing transphosphorylation and subsequent activation of downstream signaling pathways [3]. In recent years, conflicting data have emerged regarding the role of HER4 in breast cancer. Some studies suggest a negative impact of HER4 expression on disease progression, while others demonstrate beneficial effects [6]. A study involving postmenopausal breast cancer patients with varying levels of HER4 expression revealed significantly improved survival rates in those lacking HER4. These findings may be associated with the intricate interplay among these receptors [7].

There is mounting evidence of a robust association between metabolites and tumorigenesis and progression. In the context of breast cancer, in addition to the well-established pivotal roles of estrogen and progesterone in its development, an increasing body of literature has identified a diverse array of metabolites intricately linked to breast cancer [8–10]. A previous study systematically characterized metabolites in triple-negative breast cancer (TNBC) by profiling the polar metabolome and lipidome in 330 TNBC samples and 149 paired normal breast tissues, highlighting key subtype-specific metabolites as potential therapeutic targets [11]. Therefore, delving into the causal relationship between metabolites and breast cancer in depth is of significant scientific interest, especially considering the wealth of metabolomics data available and the opportunity for causal analysis among different subtypes of breast cancer characterized by distinct HER status.

Mendelian randomization (MR), a method grounded in the principles of Mendelian inheritance, serves as an indispensable analytical tool for inferring causal relationships in epidemiological studies [12]. Given the complex roles that different HER statuses play in the development and progression of breast cancer, there is a pressing need for a more comprehensive exploration. Accordingly, this study employs a comprehensive two-sample MR analysis to establish causal associations between metabolites and HER-positive/negative (HER+/−) breast cancer. The primary objective is to provide a nuanced understanding that can effectively inform clinical practices.

## METHODS

We employed a two-sample MR approach utilizing publicly available datasets that provide genome-wide association outcomes for metabolic factors, HER+ breast cancer and HER- breast cancer. Two-sample MR involves the use of distinct datasets or samples to establish the gene–risk factor associations (e.g., blood metabolites and metabolite ratios traits) and the gene–outcome associations (e.g., malignant neoplasm of breast, HER-positive/malignant neoplasm of breast, HER-negative).

### Study design

**Figure 1:**
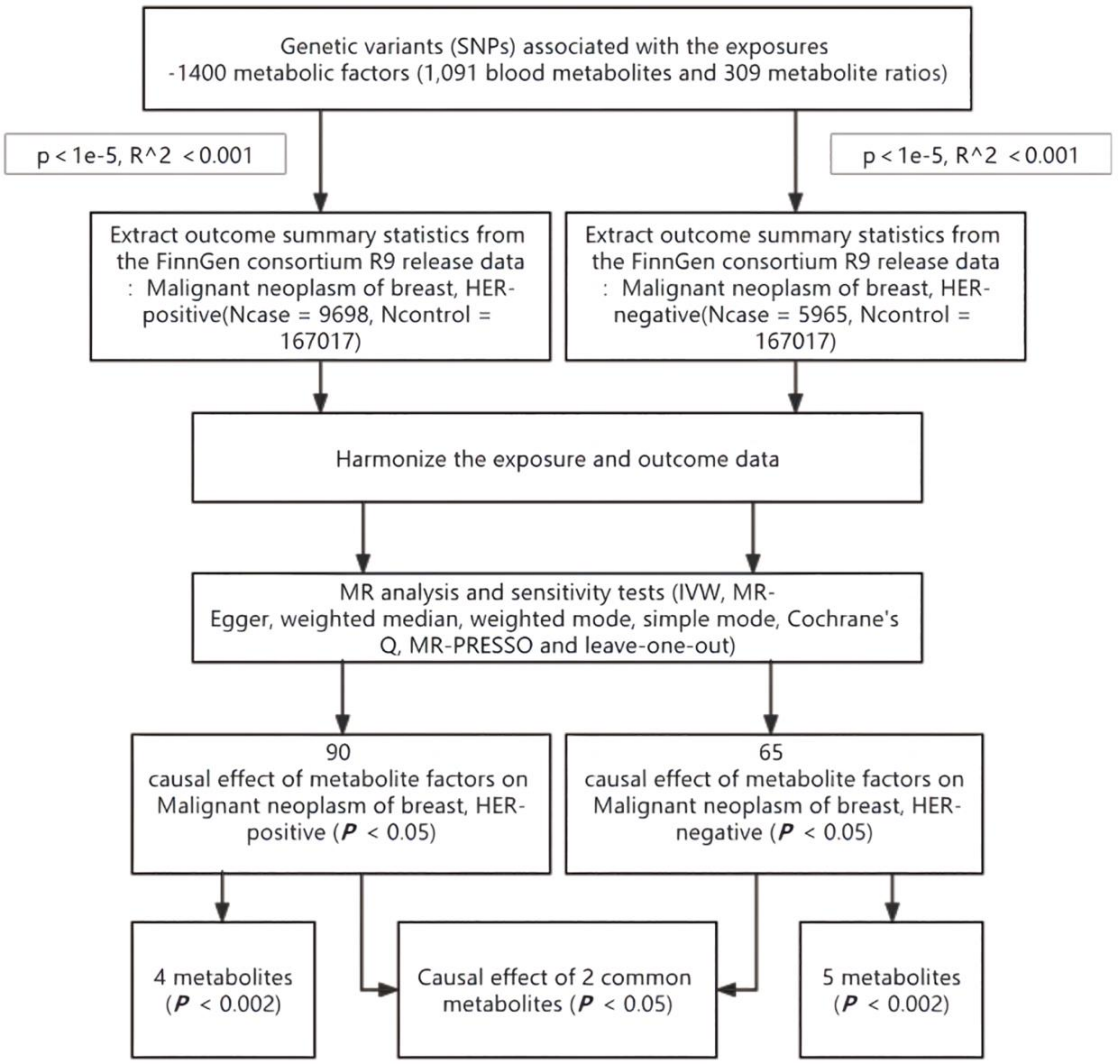
**Flow chart for study.** Abbreviations: MR: mendelian randomization; IVW: inverse-variance weighted; MR-PRESSO: MR pleiotropy residual sum and outlier.

We assessed the causal relationship between 1,400 blood metabolic factors (1,091 blood metabolites and 309 metabolite ratios) and HER+/HER− breast cancer based on a two-sample MR analysis. MR utilizes genetic variation to represent risk factors, and therefore, valid instrumental variables (IVs) in causal inference must satisfy three key assumptions: (1) genetic variation is directly associated with exposure; (2) genetic variation is not associated with possible confounders between exposure and outcome; and (3) genetic variation does not affect outcome through pathways other than exposure [13, 14]. The studies included in our analysis were approved by the relevant institutional review boards (Figure 1).

### Genome-wide association study (GWAS) data sources for HER+/HER− breast cancer

The GWAS summary statistics of HER+ breast cancer was obtained from the FinnGen consortium R9 release data. The study performed a GWAS on 176715 European individuals (Ncase = 9698, Ncontrol = 167017). As well as HER- breast cancer which performed a GWAS on 172982 European individuals (Ncase = 5965, Ncontrol = 167017).

### Metabolites GWAS data sources

GWAS summary statistics for each blood metabolic factor are publicly available from the GWAS Catalog (accession numbers from GCST90199621 to GCST90201020) [15]. A total of 1400 metabolic factors (1,091 blood metabolites and 309 metabolite ratios) were involved. The original GWAS on blood metabolites was performed using data from 8096 unrelated European subjects in Canadian Longitudinal Study of Aging (CLSA) who have been genome-wide genotyped and have had circulating plasma metabolites measured [15, 16]. Approximately there are 15.4 million single nucleotide polymorphisms (SNPs) with a minor allele frequency (MAF) higher than 0.1%, imputation quality score >0.3, and missing rate <0.1 for GWAS testing [15].

### Selection of instrumental variables (IVs)

In accordance with recent research [17, 18], the significance level of IVs for each metabolite was set to 1 × 10^−5^. To mitigate potential bias arising from strong linkage disequilibrium (LD), we implemented a clumping algorithm with a cutoff of r^2^ < 0.001 and a distance of 10,000 base pairs (kb) to ensure independence among the included SNPs. For consistency, we harmonized exposures and outcomes in terms of the effect allele and carried out subsequent analyses using the merged exposure-outcome dataset. The F statistic is a measure of instrument strength that is related to the proportion of variance in the phenotype explained by the genetic variants, sample size, and the number of instruments. An F statistic of ≥10 indicates a relatively low risk of weak instrument bias in MR analysis [19].

### Statistical analysis

To evaluate the causal association between 1400 metabolic factors and HER+/HER− breast cancer, we utilized five well-established MR methods, comprising inverse-variance weighted (IVW), MR-Egger regression, weighted median, weighted mode, and simple mode, to analyze data involving multiple IVs [20, 21]. We did not correct for multiple testing in this exploratory study. The primary emphasis was placed on the IVW method for our main results at significance of 0.05 level [22, 23], with the other methods providing supplementary insights. To gauge the heterogeneity among IVs, we employed Cochrane’s Q-statistic, considering *p* < 0.05 as indicative of significant heterogeneity [24]. If the null hypothesis is rejected, random effects IVW was used instead of fixed-effects IVW [24, 25]. In the presence of notable pleiotropy, we conducted the MR-Egger intercept test and MR pleiotropy residual sum and outlier (MR-PRESSO) method to assess directional pleiotropy [26, 27]. Furthermore, MR-PRESSO method was utilized to exclude possible horizontal pleiotropic outliers that could substantially affect the estimation results [28]. To assess result stability, we conducted a leave-one-out sensitivity analysis, systematically excluding individual IVs one at a time [29]. In addition, scatter plots and funnel plots were used. Scatter plots showed that the results were not affected by outliers. Funnel plots demonstrated the robustness of the correlation and no heterogeneity. All statistical analyses were conducted with R software (version 4.3.0) using the “TwoSampleMR” and “MR-PRESSO” packages.

### Data availability statement

All data are publicly available.

## RESULTS

### Exploration of the causal effect of metabolites on HER+ breast cancer

**Figure 2:**
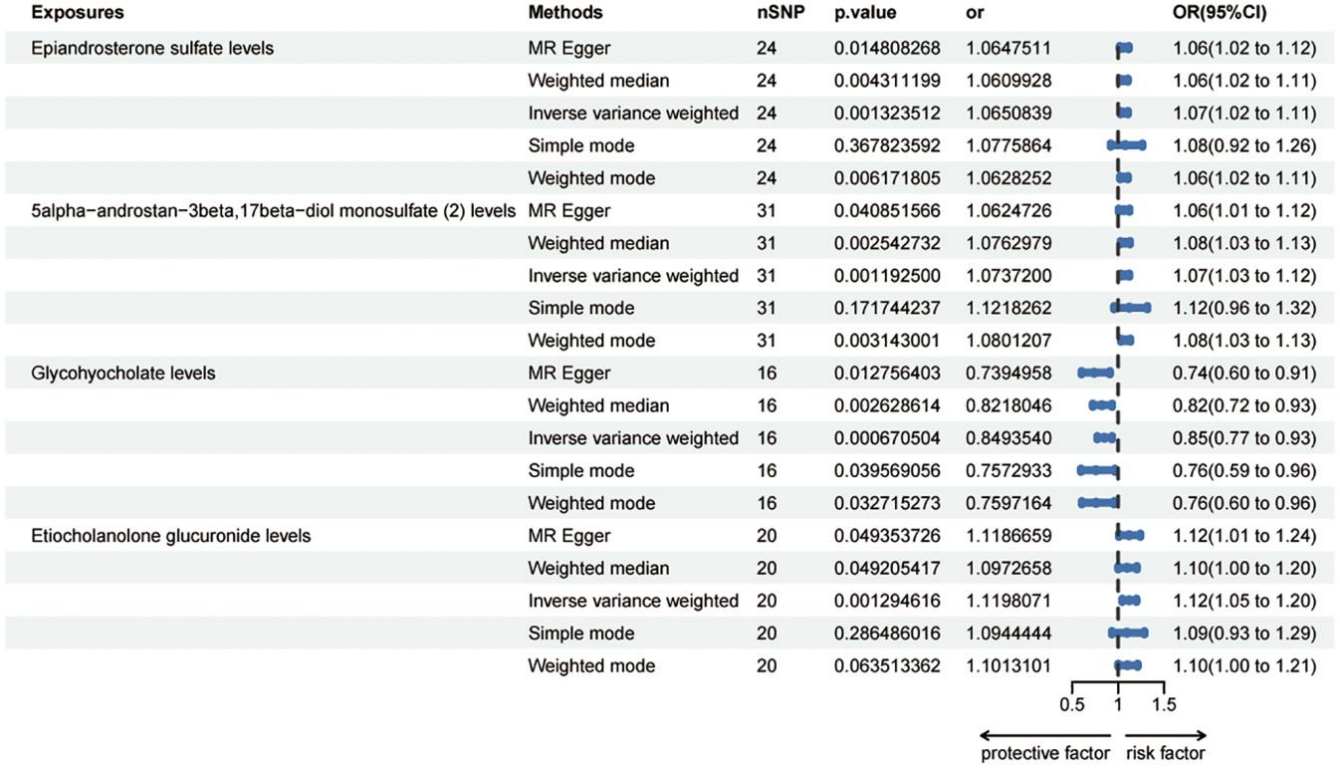
**Forest plots showed the causal associations between blood metabolites and HER+ breast cancer by using different methods.** Abbreviations: SNP: single nucleotide polymorphism; OR: odds ratio; CI: confidence interval.

The IVW method showed evidence to support that ninety metabolites were identified at a significance of 0.05, so set the *p*-value to 0.002 and detected risk effects of three metabolites on HER+ breast cancer: Epiandrosterone sulfate levels, 5alpha-androstan-3beta,17beta-diol monosulfate (2) levels and Etiocholanolone glucuronide levels. One metabolite was detected as protective effect: Glycohyocholate levels (Figure 2). The odds ratio (OR) of Epiandrosterone sulfate levels on HER+ breast cancer was estimated to be 1.07 (95% CI = 1.02 ~ 1.10, *p* = 0.0013, Supplementary Table 1) by using the IVW method. Similar results were observed by using three more methods: MR Egger (OR =1.06, 95% CI = 1.01 ~ 1.11, *p* = 0.0148), weighted median (OR = 1.06, 95% CI = 1.02 ~ 1.11, *p* = 0.0043) and weighted mode (OR = 1.06, 95% CI = 1.02 ~ 1.11, *p* = 0.0061). However, except the simple mode (OR = 0.98, 95% CI = 0.95 ~ 1.02, *p* = 0.3877). The OR of 5alpha-androstan-3beta,17beta-diol monosulfate (2) levels on HER+ breast cancer was estimated to be 1.07 (95% CI = 1.03 ~ 1.12, *p* = 0.0012, Supplementary Table 2) by using the IVW method. Similar results were observed by using three more methods: MR Egger (OR =1.06, 95% CI = 1.01 ~ 1.12, *p* = 0.0409), weighted median (OR = 1.08, 95% CI = 1.03 ~ 1.13, *p* = 0.0025) and weighted mode (OR = 1.08, 95% CI = 1.03 ~ 1.13, *p* = 0.0031). But the simple mode (OR = 1.12, 95% CI = 0.96 ~ 1.32, *p* = 0.1717) did not support this association. The OR of Glycohyocholate levels on HER+ breast cancer was estimated to be 0.85 (95% CI = 0.77 ~ 0.93, *p* = 0.0007, Supplementary Table 3) by using the IVW method. Similar results were observed by using four more methods: MR Egger (OR = 0.74, 95% CI = 0.60 ~ 0.91, *p* = 0.0128), weighted median (OR = 0.82, 95% CI = 0.72 ~ 0.94, *p* = 0.0026), simple mode (OR = 0.76, 95% CI = 0.59 ~ 0.96, *p* = 0.0396) and weighted mode (OR = 0.76, 95% CI = 0.60 ~ 0.96, *p* = 0.0327). The OR of Etiocholanolone glucuronide levels on HER+ breast cancer was estimated to be 1.12 (95% CI = 1.05 ~ 1.20, *p* = 0.0013, Supplementary Table 4) by using the IVW method. Similar results were observed by using two more methods: MR Egger (OR = 1.19, 95% CI = 1.01 ~ 1.24, *p* = 0.0494) and weighted median (OR = 1.10, 95% CI = 1.00 ~ 1.20, *p* = 0.0492). However, simple mode (OR = 1.09, 95% CI = 0.93 ~ 1.29, *p* = 0.2865) and weighted mode (OR = 1.10, 95% CI = 1.00 ~ 1.21, *p* = 0.0635) did not support this association. These trends are also evident in the forest plots (Supplementary Figure 1A–1D) and scatter plots (Supplementary Figure 2A–2D).

### Exploration of the causal effect of metabolites on HER- breast cancer

**Figure 3:**
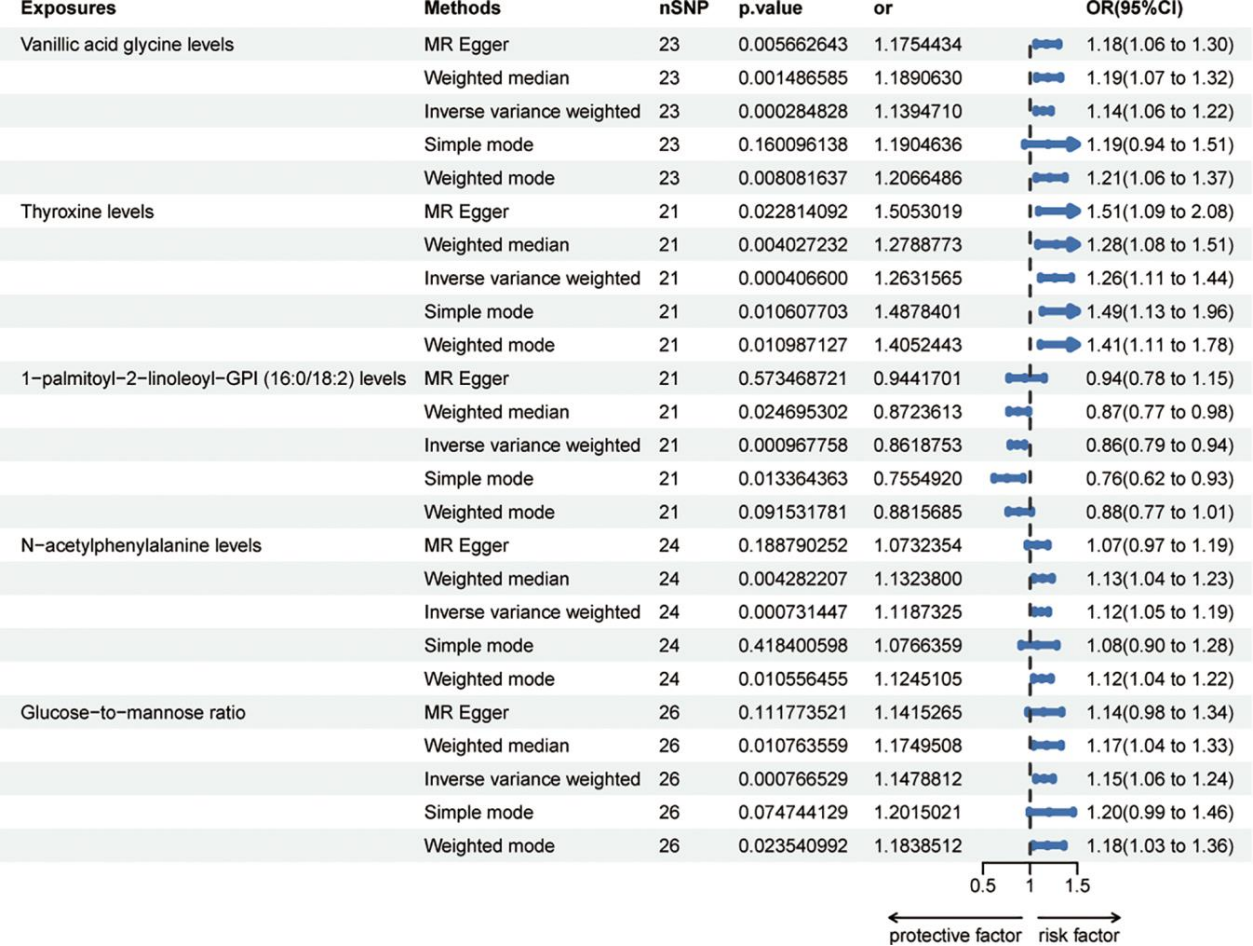
**Forest plots showed the causal associations between blood metabolites and HER- breast cancer by using different methods.** Abbreviations: SNP: single nucleotide polymorphism; OR: odds ratio; CI: confidence interval.

Sixty-five metabolites were identified to be significant at a *p*-value of 0.05 using the IVW method; subsequently, setting the *p*-value to 0.002 revealed the risk effects of four metabolites on HER-negative breast cancer: Vanillic acid glycine levels, Thyroxine levels, N-acetylphenylalanine levels and Glucose-to-mannose ratio. Meanwhile, 1-palmitoyl-2-linoleoyl-GPI (16:0/18:2) levels was detected as a protective effector (Figure 3). The odds ratio (OR) for Vanillic acid glycine levels and the risk of HER- breast cancer was estimated to be 1.14 (95% CI = 1.06 ~ 1.22, *p* = 0.0003, Supplementary Table 5) using the IVW method. Consistent results were obtained with three other methods: MR Egger (OR = 1.18, 95% CI = 1.06 ~ 1.30, *p* = 0.0057), weighted median (OR = 1.19, 95% CI = 1.07 ~ 1.32, *p* = 0.0015), and weighted mode (OR = 1.21, 95% CI = 1.06 ~ 1.37, *p* = 0.0080). However, the simple mode did not provide support for this association (OR = 1.19, 95% CI = 0.94 ~ 1.51, *p* = 0.1601). Thyroxine levels showed an OR of 1.26 (95% CI = 1.11 ~ 1.44, *p* = 0.0004, Supplementary Table 6) for HER- breast cancer risk using the IVW method. Similar results were observed with four additional methods: MR Egger (OR = 1.51, 95% CI = 1.09 ~ 2.08, *p* = 0.0228), weighted median (OR = 1.28, 95% CI = 1.08 ~ 1.51, *p* = 0.0040), simple mode (OR = 1.49, 95% CI = 1.13 ~ 1.96, *p* = 0.0106), and weighted mode (OR = 1.41, 95% CI = 1.11 ~ 1.78, *p* = 0.0110). For 1-palmitoyl-2-linoleoyl-GPI (16:0/18:2) levels, the OR for HER- breast cancer risk was estimated as 0.86 (95% CI = 0.79 ~ 0.94, *p* = 0.0010, Supplementary Table 7) using the IVW method. The weighted median (OR = 0.87, 95% CI = 0.77 ~ 0.98, *p* = 0.0247) and simple mode (OR = 0.75, 95% CI = 0.62 ~ 0.93, *p* = 0.0134) also supported this association. However, the MR Egger (OR = 0.94, 95% CI = 0.78 ~ 1.15, *p* = 0.5735) and weighted mode (OR = 0.88, 95% CI = 0.77 ~ 1.01, *p* = 0.0915) did not find evidence to support this relationship. N-acetylphenylalanine levels exhibited an OR of 1.12 (95% CI = 1.05 ~ 1.19, *p* = 0.0007, Supplementary Table 8) for HER- breast cancer risk using the IVW method. Consistent results were obtained with the weighted median (OR = 1.13, 95% CI = 1.04 ~ 1.23, *p* = 0.0043) and weighted mode (OR = 1.12, 95% CI = 1.04 ~ 1.22, *p* = 0.0106). However, the MR Egger (OR = 1.07, 95% CI = 0.97 ~ 1.19, *p* = 0.1888) and simple mode did not support this association (OR = 1.08, 95% CI = 0.90 ~ 1.28, *p* = 0.4184). The OR for Glucose-to-mannose ratio and HER- breast cancer risk was estimated as 1.15 (95% CI = 1.06 ~ 1.24, p = 0.0008, Supplementary Table 9) using the IVW method. Consistent results were observed with the weighted median (OR = 1.17, 95% CI = 1.04 ~ 1.33, *p* = 0.0108) and weighted mode (OR = 1.18, 95% CI = 1.03 ~ 1.36, *p* = 0.0235). However, the MR Egger (OR = 1.14, 95% CI = 0.98 ~ 1.34, *p* = 0.1118) and simple mode (OR = 1.20, 95% CI = 0.99 ~ 1.46, *p* = 0.0747) did not support this association. These associations are also noticeable in both the forest plots (Supplementary Figure 1E–1I) and scatter plots (Supplementary Figure 2E–2I).

### Exploration of the causal effect of intersecting metabolites on both HER+ and HER- breast cancer

**Figure 4:**
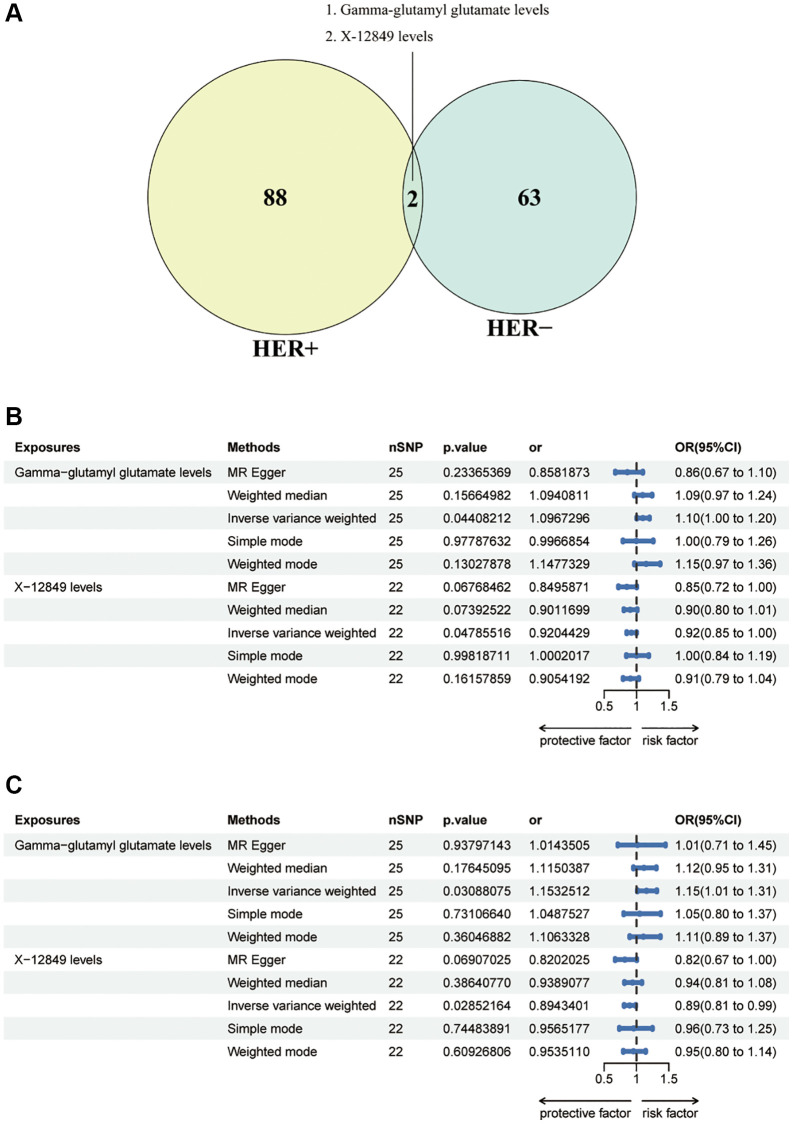
(**A**) Venn diagram showed the intersection of blood metabolites that are causally involved in HER+ and HER-breast cancers. (**B**) Forest plots showed the causal associations between blood metabolites (Gamma-glutamyl glutamate levels and X-12849 levels) and HER+ breast cancer by using different methods. (**C**) Forest plots showed the causal associations between blood metabolites (Gamma-glutamyl glutamate levels and X-12849 levels) and HER- breast cancer by using different methods. Abbreviations: SNP: single nucleotide polymorphism; OR: odds ratio; CI: confidence interval.

In order to examine the causal effects of common metabolites on both HER+ and HER– breast cancer, we identified a total of 90 metabolites associated with HER+ breast cancer and 65 metabolites associated with HER- breast cancer at a significance level of 0.05 using the IVW method as the primary analysis. By taking the intersection of these sets, we detected two metabolites that were consistently present in both HER+ and HER- breast cancer: Gamma-glutamyl glutamate levels and X-12849 levels (Figure 4A). Interestingly, Gamma-glutamyl glutamate levels were found to act as risk effectors in both HER+ and HER- breast cancer, while X-12849 levels exhibited a protective effect (Figure 4B, 4C). The association between Gamma-glutamyl glutamate levels and HER+ breast cancer risk was estimated to have an odds ratio (OR) of 1.26 (95% CI = 0.67 ~ 1.10, *p* = 0.0004) using the IVW method. However, other methods such as MR Egger (OR = 0.86, 95% CI = 1.09 ~ 2.08, *p* = 0.2337), weighted median (OR = 1.09, 95% CI = 0.97 ~ 1.24, *p* = 0.1567), simple mode (OR = 0.99, 95% CI = 0.79 ~ 1.26, *p* = 0.9779), and weighted mode (OR = 1.15, 95% CI = 0.97 ~ 1.36, *p* = 0.1303) did not support this association (Supplementary Table 10). The MR Egger analysis, weighted median, simple mode, and weighted mode did not provide evidence for a causal relationship. Similarly, the OR of X-12849 levels on HER+ breast cancer risk was estimated as 0.92 (95% CI = 0.85 ~ 1.00, *p* = 0.0479) using the IVW method. However, the MR Egger (OR = 0.85, 95% CI = 0.72 ~ 1.00, *p* = 0.0677), weighted median (OR = 0.90, 95% CI = 0.80 ~ 1.01, *p* = 0.0739), simple mode (OR = 1.00, 95% CI = 0.84 ~ 1.19, *p* = 0.9982), and weighted mode (OR = 0.91, 95% CI = 0.79 ~ 1.04, *p* = 0.1616) did not support this association (Supplementary Table 11). Regarding HER- breast cancer risk, the OR of Gamma-glutamyl glutamate levels was estimated as 1.15 (95% CI = 1.01 ~ 1.31, *p* = 0.0309) using the IVW method. However, the MR Egger (OR = 1.01, 95% CI = 0.71 ~ 1.45, *p* = 0.9380), weighted median (OR = 1.12, 95% CI = 0.95 ~ 1.31, *p* = 0.1765), simple mode (OR = 1.05, 95% CI = 0.80 ~ 1.37, *p* = 0.7311), and weighted mode (OR = 1.11, 95% CI = 0.89 ~ 1.37, *p* = 0.3605) did not support this association (Supplementary Table 12). Similarly, the OR of X-12849 levels on HER- breast cancer risk was estimated as 0.89 (95% CI = 0.81 ~ 0.99, *p* = 0.0285) using the IVW method. However, the MR Egger (OR = 0.82, 95% CI = 0.67 ~ 1.00, *p* = 0.0691), weighted median (OR = 0.94, 95% CI = 0.81 ~ 1.08, *p* = 0.3864), simple mode (OR = 0.96, 95% CI = 0.73 ~ 1.25, *p* = 0.7448), and weighted mode (OR = 0.95, 95% CI = 0.80 ~ 1.14, *p* = 0.6093) did not support this association (Supplementary Table 13). These results could also be observed in the forest plots and scatter plots (Supplementary Figure 3).

### Sensitivity analysis

We conducted multiple sensitivity analyses to assess the presence of heterogeneity and pleiotropy in our causal estimates. Cochran’s *Q*-test and MR-PRESSO test indicated no significant heterogeneity or pleiotropy among the SNPs involved in the causal relationships (Supplementary Tables 14–16). Funnel plots exhibited symmetrical distribution, suggesting no evidence of publication bias across these analyses (Supplementary Figures 4, 5A–5D). Furthermore, the sensitivity analysis, performed through leave-one-out analysis, confirmed the robustness of the causal associations (Supplementary Figures 6, 5E–5H).

## DISCUSSION

In this study, we conducted separate analyses to investigate the causal associations between 1400 metabolic factors, comprising 1,091 blood metabolites and 309 metabolite ratios, and HER+ and HER– breast cancer. We utilized large publicly available genetic data to explore these associations comprehensively. By examining a wide range of metabolic factors, including individual metabolites and their ratios, we aimed to gain insights into the potential causal relationships between these factors and breast cancer subtypes. Due to the large number of metabolites identified at a significance level of *p* < 0.05 in the result, we made the decision to narrow down our focus by selecting only the four to five metabolites with the smallest *p*-values (*p* < 0.002) within each isoform for detailed presentation and discussion. This approach allows us to concentrate on the most statistically significant metabolites and facilitate a more targeted analysis. The findings of our study reveal several significant associations with respect to the causal risk factors for different breast cancer subtypes. For HER+ breast cancer, elevated levels of epiandrosterone sulfate, 5alpha-androstan-3beta,17beta-diol monosulfate (2), and etiocholanolone glucuronide were found to be causally associated with an increased risk. Conversely, increased glycohyocholate levels were found to be causally associated with a decreased risk for HER- breast cancer. In the case of HER- breast cancer, we identified several causal risk factors. These include elevated levels of vanillic acid glycine, thyroxine, N-acetylphenylalanine, and a higher glucose-to-mannose ratio. On the other hand, we observed that increased levels of 1-palmitoyl-2-linoleoyl-GPI (16:0/18:2) were causally associated with a decreased risk for HER- breast cancer. Furthermore, when considering the intersection of both breast cancer subtypes, we found a causal association between elevated levels of Gamma-glutamyl glutamate and an increased risk for both HER+ and HER– breast cancer. Additionally, increased levels of X-12849 were causally associated with a decreased risk for both subtypes.

Metabolomics analysis currently relies on a range of detection techniques, notably Liquid Chromatography with tandem mass spectrometry (LC-MS/MS) and others. These methodologies have identified numerous metabolites linked to tumor development, which are being extensively studied as potential therapeutic targets [30, 31]. A prospective study revealed a strong correlation between plasma concentrations of metabolites and breast cancer risk. Specifically, concentrations of arginine, asparagine, and phosphatidylcholine were found to be negatively associated with breast cancer risk, while acylcarnitines exhibited a positive association [31]. In the quest for diagnostic biomarkers in early breast cancer, Wei et al. leveraged untargeted liquid chromatography quadrupole time-of-flight mass spectrometry (LC-QTOF-MS) data to uncover a spectrum of promising metabolites including ethyl (R)-3-hydroxyhexanoate, caprylic acid, hypoxanthine, and others [32]. These findings have provided valuable insights into the potential pathogenesis of early-stage breast cancer. Aligned with this objective, our study also aimed to elucidate metabolites that play a causal role in breast carcinogenesis.

Among the metabolites causally linked to HER+ breast cancer, epiandrosterone sulfate and 5alpha-androstan-3beta-17beta-diol play crucial roles in androgen metabolism [33, 34]. Androgens have been extensively researched and validated for their involvement in various tumorigenesis processes. However, the specific relationships between epiandrosterone sulfate, 5alpha-androstan-3beta-17beta-diol monosulfate, and the risk of HER+ breast cancer remain inconclusive and warrant further investigation. Understanding the significant impact of androgen excess on diverse breast cancer subtypes holds substantial clinical implications for treatment and prevention [35]. Therefore, epiandrosterone sulfate and 5alpha-androstan-3beta-17beta-diol monosulfate show promise as potential therapeutic targets for HER+ breast cancer. Etiocholanolone glucuronide (Etio-G) is a primary testosterone metabolite, alongside androsterone glucuronide (ADT-G), Testosterone glucuronide (TG), and dihydrotestosterone glucuronide (DHTG). The liver and intestines are key sites for Etio-G formation, which is subsequently released into the bloodstream [36]. While previous studies indicate that elevated androsterone-glucuronide levels are linked to an increased risk of non-serous ovarian cancer, the association between Etio-G and cancer remains largely unexplored. Our findings shed new light on the relationship between Etio-G and breast cancer. Belonging to the primary bile acid (BA) family, glycohyocholate has been shown to significantly reduce the risk of nonalcoholic fatty liver disease (NAFLD) [37]. Although bile acids were traditionally viewed as pro-carcinogenic agents (e.g., esophageal cancer), recent evidence suggests that physiological concentrations of bile acids possess anti-cancer properties in certain cancers such as prostate, ovarian, and breast cancer [38]. Notably, breast cancer patients exhibit reduced hepatic bile acid production, reflected in lower serum and fecal bile acid levels. Furthermore, the transformation of bile acids into secondary forms by gut bacteria is also diminished [38–40]. Our discovery that Glycohyocholate acts protectively in HER+ breast cancer aligns with existing research and implies its potential utility as both a diagnostic tool and therapeutic target for breast cancer.

For HER- breast cancer, our study identified five metabolites with causal links. Our findings indicate that the level of vanillic acid glycine may lean towards acting as a risk factor for tumor development. The focus of current study primarily centers around vanillic acid. The impact of vanillic acid on tumors appears to be multifaceted. Zhu et al. demonstrated its potential as an antitumor agent by activating the stimulator of interferon genes (STING) signaling pathway in macrophages [41]. In colon cancer cells, vanillic acid exerts inhibitory effects on HIF-1alpha expression through the mTOR/p70S6K/4E-BP1 and Raf/MEK/ERK pathways [42]. However, Ujlaki et al. observed hyperproliferative effects when vanillic acid was administered to the mouse breast cancer cell line 4T1 [43]. Epidemiological studies have established associations between thyroid function and breast cancer, suggesting that hormones can play a supportive role in breast cancer development. L-thyroxine (T4) has been demonstrated to induce the proliferation of various types of cancer. This T4-induced activity is facilitated by a cell surface receptor located on the extracellular domain of integrin αvβ3. Subsequently, the T4 signal is transduced by mitogen-activated protein kinase (MAPK/ERK1/2) or phosphatidylinositol 3-kinase (PI3-K) pathways, leading to gene transcription associated with cancer [44]. Notably, T4 has been also identified as a proliferative factor for breast cancer cells in laboratory experiments [45, 46]. Additionally, T4 upregulates the accumulation of checkpoint programmed death-ligand 1 (PD-L1) in cancer cells [47]. However, uncertainties remain regarding whether circulating endogenous T4 levels act as a risk factor for breast cancer among individuals with normal thyroid function but a positive family history. Further research is crucial to unravel the intricate relationship between thyroid hormone levels and breast cancer risk in this specific subgroup of patients.

Currently, there is a scarcity of research on 1-palmitoyl-2-linoleoyl-GPI (16:0/18:2). However, a study by Poupore et al. delved into metabolite distinctions between patients with ischemic stroke and control subjects. In the female group, a total of 1322 biochemicals were identified, comprising 1062 named compounds with known identities and 260 unnamed compounds with unidentified structural features. Notably, among these compounds, 1-palmitoyl-2-linoleoyl-GPI (16:0/18:2) displayed significant differences and might hold promise as a diagnostic indicator for ischemic stroke [48]. Our own research findings suggest that 1-palmitoyl-2-linoleoyl-GPI (16:0/18:2) functions as a protective element against HER- breast malignancies. Therefore, further investigation into this metabolite could offer a fruitful path for future exploration. While limited information exists on the association between N-acetylphenylalanine and cancer, insights from a study conducted by Tsamouri suggest potential implications. The study suggested that urinary N-acetylphenylalanine levels could serve as a diagnostic marker for uroepithelial carcinoma of the bladder in dogs [49]. Nevertheless, further investigations are necessary to fully grasp the role of N-acetylphenylalanine in cancer development and progression in humans. In our study, the Glucose-to-mannose ratio was identified as a risk factor for HER- breast cancer. This suggests that an increased glucose level or decreased mannose level would elevate the ratio. Glucose metabolism significantly supports tumor cell growth and proliferation [50]. Conversely, mannose (C6H12O6) has demonstrated tumor growth inhibition in both *in vitro* and *in vivo* studies [50]. Regarding diagnosis, the serum free glucose to mannose ratio holds promise as a potential biomarker for ovarian cancer [51]. Noteworthy results showed a 49% reduction in recurrence risk and a 56% decrease in death risk for esophageal adenocarcinoma (EAC) cases among patients with elevated mannose levels compared to those with lower levels [52, 53]. These findings underscore the potential utility of serum mannose as a diagnostic or prognostic tool for various tumor types.

According to our study, Gamma-glutamylglutamate levels and X-12849 levels are identified as common factors with a causal relationship in both HER+ and HER– breast cancer subtypes. Gamma-glutamylglutamate is a dipeptide formed by the condensation of the gamma-carboxy group of glutamic acid with the amino group of another glutamic acid. Notably, a metabolomic analysis involving 1812 Finnish men and Huang’s Cox proportional hazards regression model revealed an association between gamma-glutamylglutamate and an increased risk of prostate cancer-specific mortality [54]. However, whether Gamma-glutamylglutamate acts as a risk factor for breast cancer remains uncertain. In our investigation, we found supporting evidence for a causal link between Gamma-glutamylglutamate and both HER+ and HER– breast cancers. This suggests that Gamma-glutamylglutamate may play a significant role in breast cancer, warranting further exploration and clarification through additional studies. In the realm of untargeted metabolomics, identifying metabolites continues to pose a significant challenge. Typically, metabolites lacking a known chemical structure are denoted with the prefix “X-” followed by a number [55]. Based on our research outcomes, we observed that X-12849 levels act as protective elements against both HER+ and HER– breast carcinogenesis. These findings not only enhance our comprehension of the impact of X-12849 but also offer valuable insights into the connection between unidentified metabolites and human diseases.

Our study employed a two-sample MR analysis, utilizing data from large-scale GWAS cohorts to ensure statistical robustness. This approach allowed us to minimize the impact of confounding factors, such as horizontal pleiotropy and related variables, on our results. However, it is important to acknowledge several limitations in our study. Firstly, despite conducting multiple sensitivity analyses, fully assessing the presence of horizontal pleiotropy remains challenging. This potential source of bias should be considered when interpreting the results. One major problem is that we found evidence for pleiotropy in our MR of Gamma-glutamyl glutamate levels on HER+ breast cancer, but pleiotropy was eliminated by MR PRESSO. Secondly, due to the lack of individual-level data (e.g., stage, grade, and hormone receptor status), we were unable to perform further stratified analyses within the population. This limits our ability to draw conclusions specific to certain subgroups. Thirdly, it is important to note that in our study, we opted to use a more lenient threshold and did not correct for multiple testing when evaluating the results. While this approach aimed to maximize the detection of potential associations, it also introduces the possibility of increased false positives. Therefore, caution should be exercised when interpreting these findings, and further validation studies are necessary to confirm the observed associations. Lastly, it is worth noting that the external validity of our findings may be limited since the data source for this study primarily consisted of a European population. Generalizing the results to other populations should be done cautiously. Despite these limitations, our study provides valuable insights into the causal effects of hub metabolites on both HER+ and HER− breast cancer, highlighting the need for further research in this area.

In summary, our MR analysis has revealed significant causal relationships between various metabolites and breast cancer characterized by HER+ or HER– expression. This finding not only sheds light on the intricate interactions between metabolites and breast carcinogenesis but also advances our comprehension of the realm of breast cancer and metabolomics. Importantly, certain metabolites that have been overlooked in terms of their association with tumors indicate promising avenues for further investigation. Therefore, further research is essential to elucidate the intricate mechanisms involving metabolites in breast carcinogenesis and to evaluate the feasibility of clinical interventions. These endeavors will not only yield new insights into the origins of breast cancer but also enhance treatment strategies.

## Supplementary Materials

Supplementary Figures

Supplementary Tables
