# Supplementary material for: Causal role of blood metabolites in HER-positive and HER-negative breast cancer: a Mendelian randomization (MR) study
Source: Aging (Albany NY). 2024 Aug 2;16(15):11626–55. doi: 10.18632/aging.206042 (PMC11346783; doi:10.18632/aging.206042)
Supplement: Supplementary Figures [file aging-16-206042-s001.pdf]

SUPPLEMENTARY FIGURES

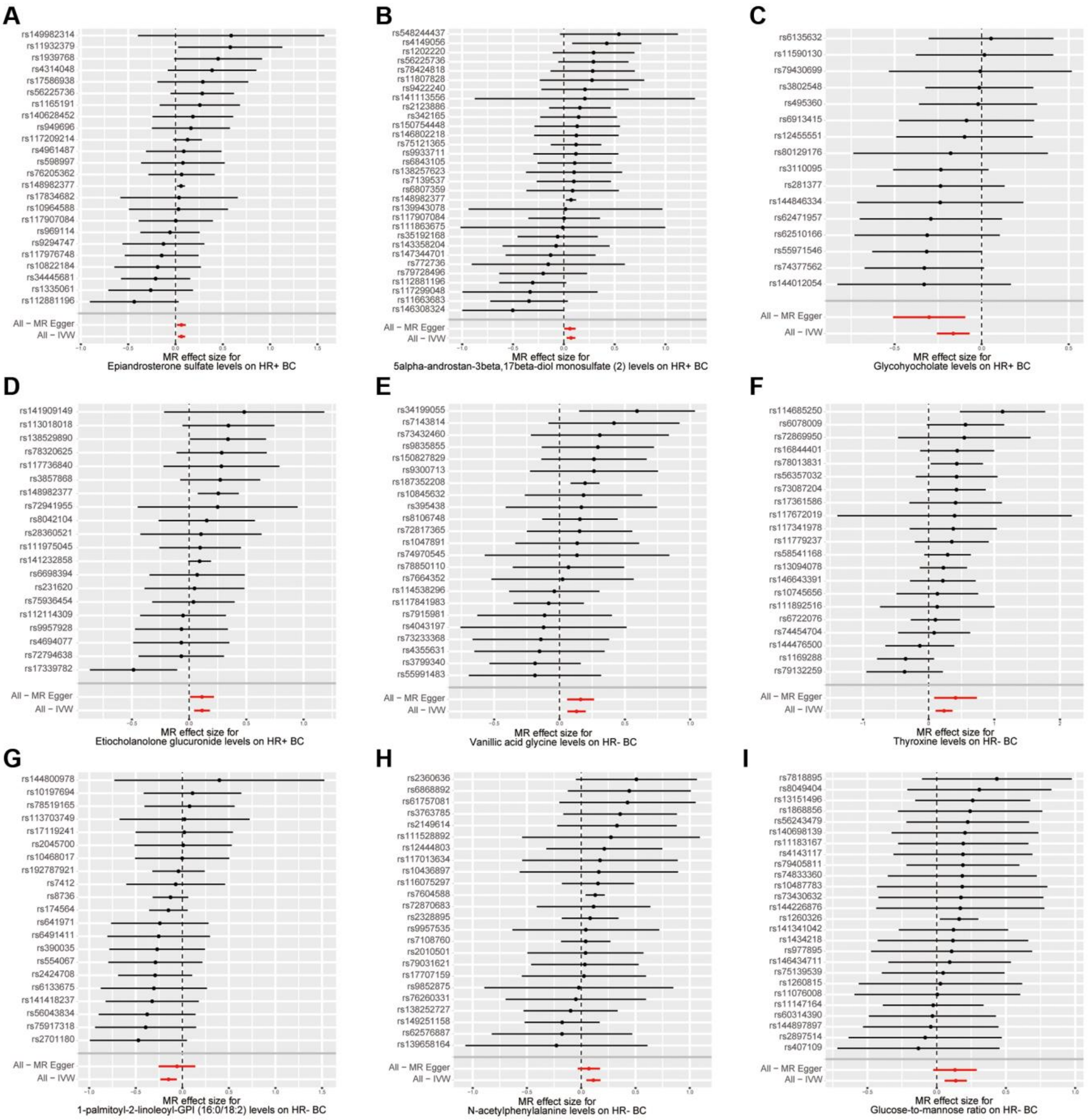

Supplementary Figure 1. Forest plots of SNPs associated with metabolic factors and the risk of HER+/HER− BC. (**A**) Epiandrosterone sulfate levels on HER+ BC. (**B**) 5alpha-androstan-3beta,17beta-diol monosulfate (2) levels on HER+ BC. (**C**) Glycohyocholate levels on HER+ BC. (**D**) Etiocholanolone glucuronide levels on HER+ BC. (**E**) Vanillic acid glycine levels on HER- BC. (**F**) Thyroxine levels on HER- BC. (**G**) 1-palmitoyl-2-linoleoyl-GPI (16:0/18:2) levels on HER-BC. (**H**) N-acetylphenylalanine levels on HER- BC. (**I**) Glucose-to-mannose ratio on HER- BC. Abbreviations: SNP: single nucleotide polymorphisms; HER: human epidermal growth factor receptor; BC: breast cancer; IVW: inverse-variance weighted.

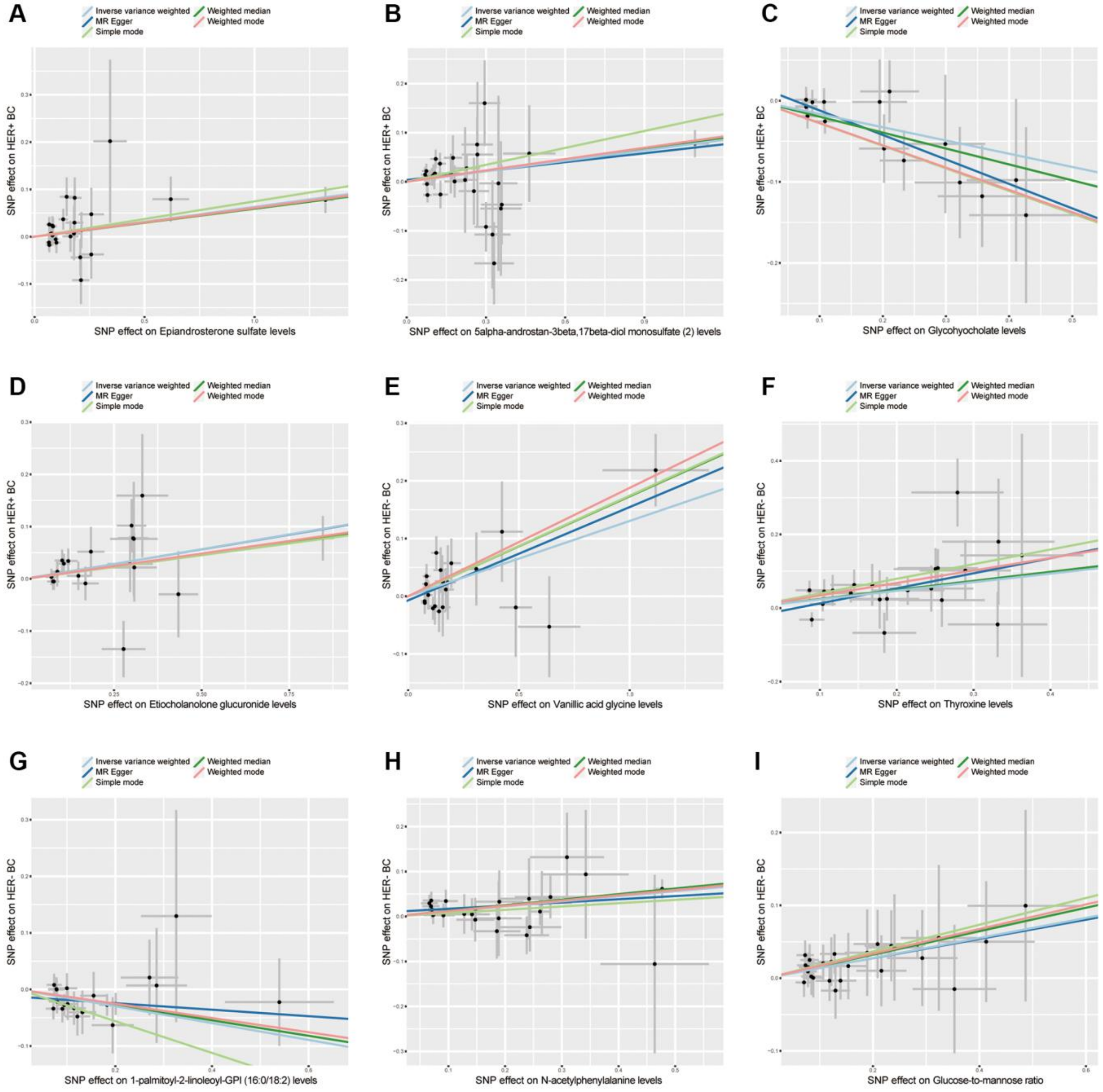

Supplementary Figure 2. Scatter plots showed the genetic associations of metabolic factors and HER+/HER− BC. (**A**) Epiandrosterone sulfate levels on HER+ BC. (**B**) 5alpha-androstan-3beta,17beta-diol monosulfate (2) levels on HER+ BC. (**C**) Glycohyocholate levels on HER+ BC. (**D**) Etiocholanolone glucuronide levels on HER+ BC. (**E**) Vanillic acid glycine levels on HER- BC. (**F**) Thyroxine levels on HER- BC. (**G**) 1-palmitoyl-2-linoleoyl-GPI (16:0/18:2) levels on HER-BC. (**H**) N-acetylphenylalanine levels on HER- BC. (**I**) Glucose-to-mannose ratio on HER- BC. Abbreviations: SNP: single nucleotide polymorphisms; HER: human epidermal growth factor receptor; BC: breast cancer.

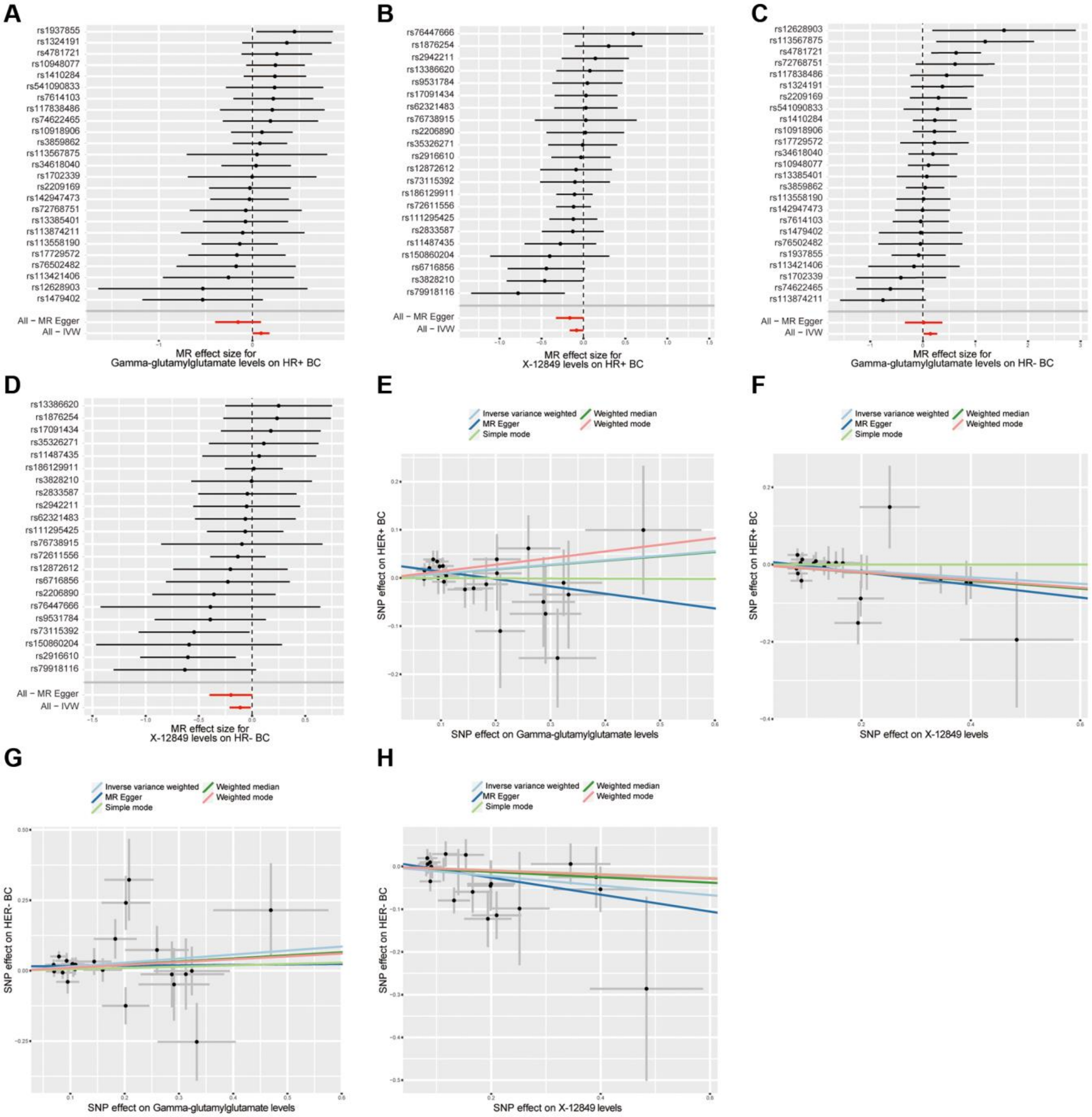

Supplementary Figure 3. Forest plots and scatter plots of metabolic factors on HER+/HER− BC. (**A**) Forest plot of Gamma-glutamylglutamate levels on HER+ BC. (**B**) Forest plot of X-12849 levels on HER+ BC. (**C**) Forest plot of Gamma-glutamylglutamate levels on HER- BC. (**D**) Forest plot of X-12849 levels on HER- BC. (**E**) Scatter plot of Gamma-glutamylglutamate levels on HER+ BC. (**F**) Scatter plot of X-12849 levels on HER+ BC. (**G**) Scatter plot of Gamma-glutamylglutamate levels on HER- BC. (**H**) Scatter plot of X-12849 levels on HER- BC. Abbreviations: SNP: single nucleotide polymorphisms; HER: human epidermal growth factor receptor; BC: breast cancer.

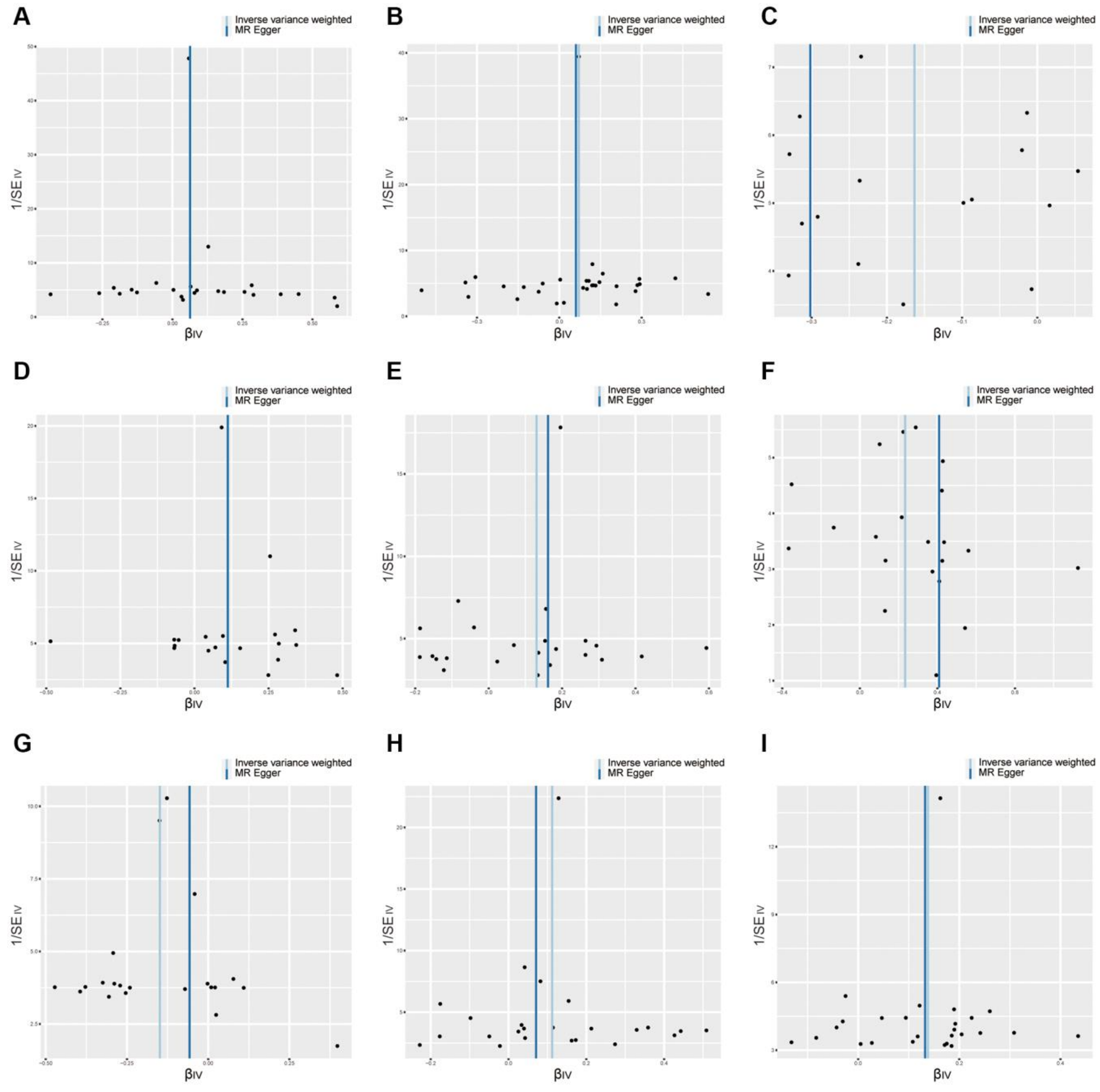

Supplementary Figure 4. Funnel plots showed the heterogeneity between metabolic factors and HER+/HER− BC. (**A**) Epiandrosterone sulfate levels on HER+ BC. (**B**) 5alpha-androstan-3beta,17beta-diol monosulfate (2) levels on HER+ BC. (**C**) Glycohyocholate levels on HER+ BC. (**D**) Etiocholanolone glucuronide levels on HER+ BC. (**E**) Vanillic acid glycine levels on HER- BC. (**F**) Thyroxine levels on HER- BC. (**G**) 1-palmitoyl-2-linoleoyl-GPI (16:0/18:2) levels on HER-BC. (**H**) N-acetylphenylalanine levels on HER- BC. (**I**) Glucose-to-mannose ratio on HER- BC. Abbreviations: Beta: risk index; Se: standard error.

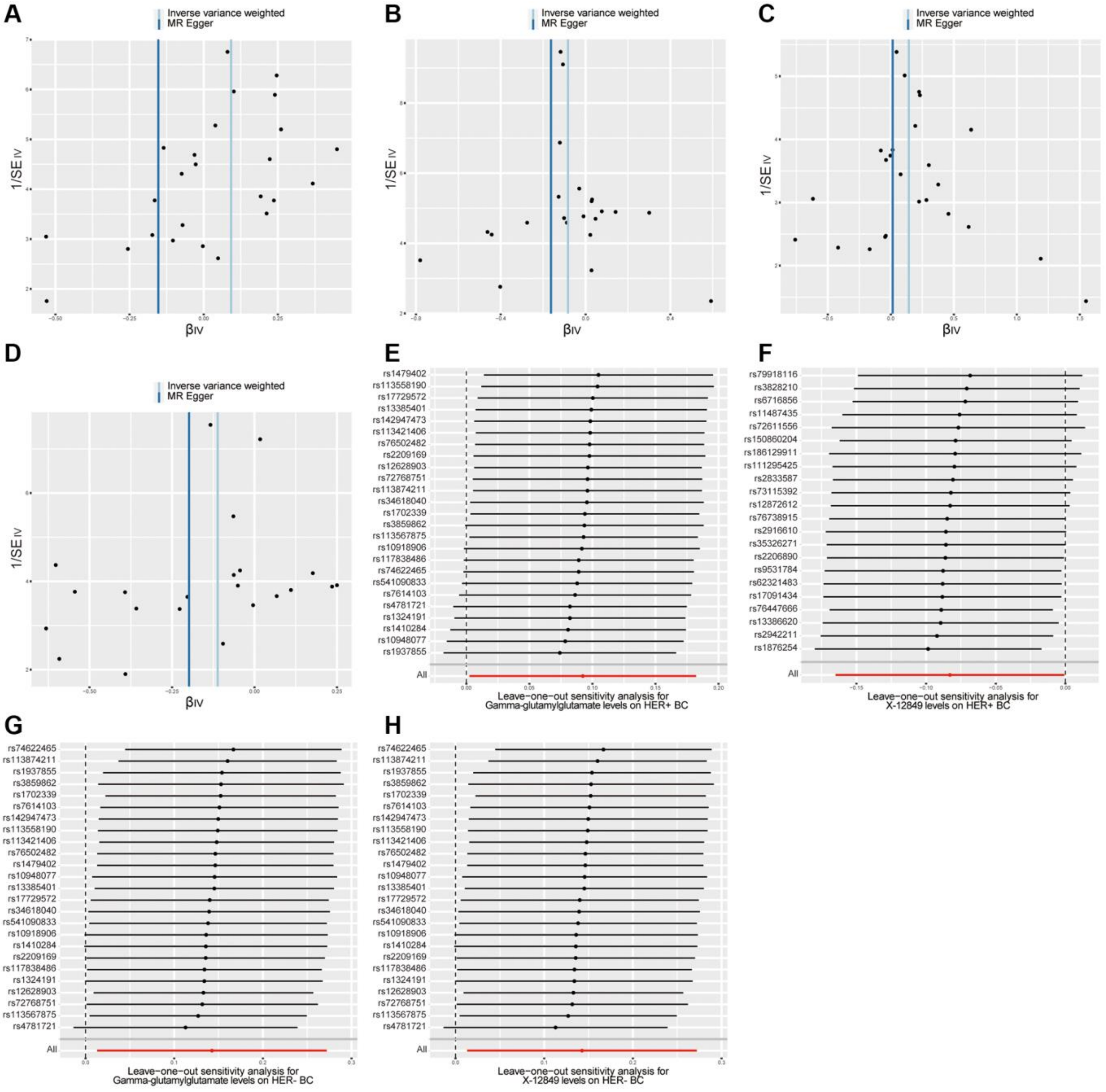

Supplementary Figure 5. Funnel plots and leave-one-out plots of metabolic factors on HER+/HER− BC. (**A**) Funnel plot of Gamma-glutamylglutamate levels on HER+ BC. (**B**) Funnel plot of X-12849 levels on HER+ BC. (**C**) Funnel plot of Gamma-glutamylglutamate levels on HER-BC. (**D**) Funnel plot of X-12849 levels on HER- BC. (**E**) Leave-one-out plot of Gamma-glutamylglutamate levels on HER+ BC. (**F**) Leave-one-out plot of X-12849 levels on HER+ BC. (**G**) Leave-one-out plot of Gamma-glutamylglutamate levels on HER- BC. (**H**) Leave-one-out plot of X-12849 levels on HER- BC. Abbreviations: SNP: single nucleotide polymorphisms; HER: human epidermal growth factor receptor; BC: breast cancer.

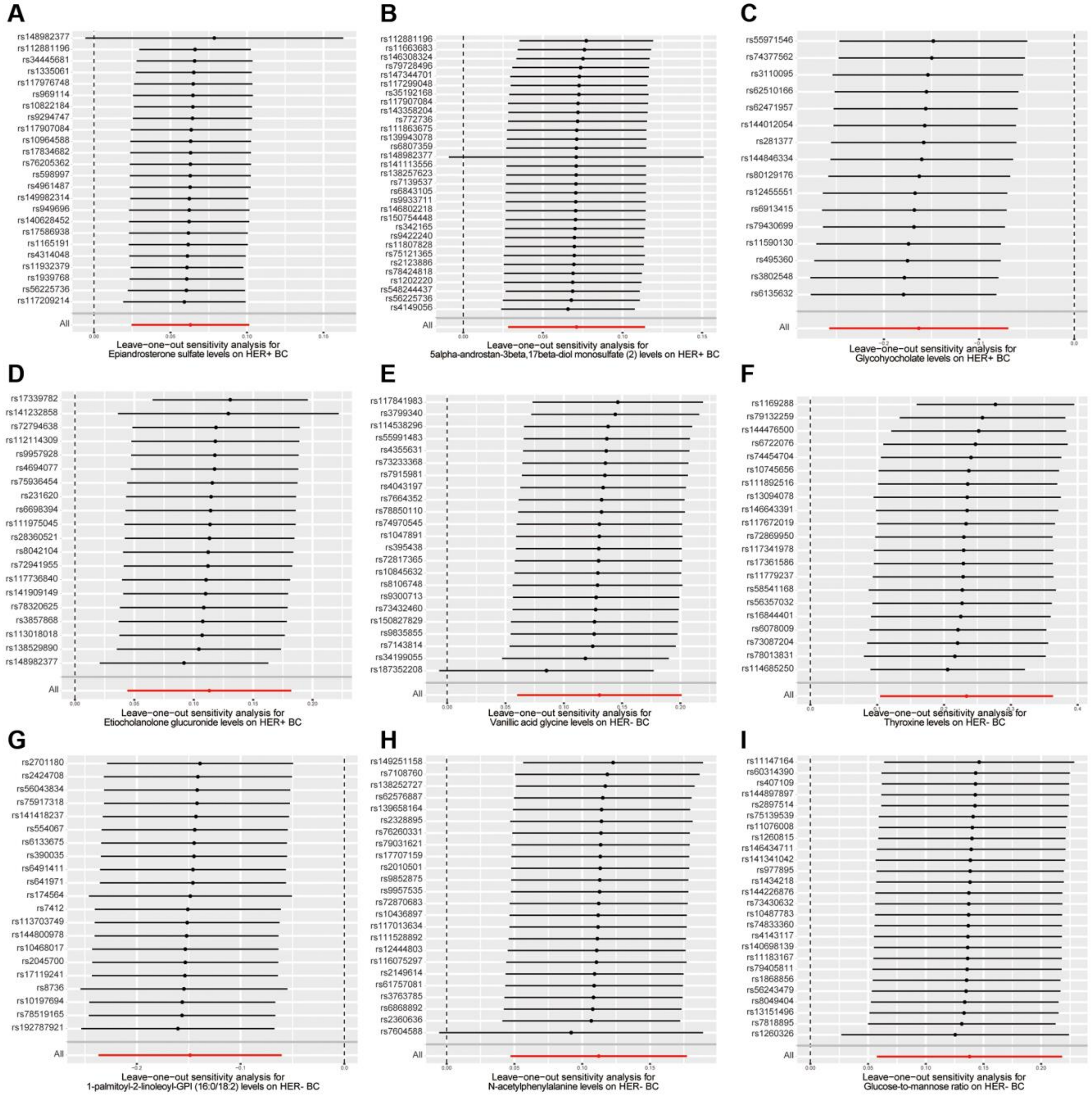

Supplementary Figure 6. Leave-one-out plots for the causal effects of metabolic factors on HER+/HER− BC. (**A**) Epiandrosterone sulfate levels on HER+ BC. (**B**) 5alpha-androstan-3beta,17beta-diol monosulfate (2) levels on HER+ BC. (**C**) Glycohyocholate levels on HER+ BC. (**D**) Etiocholanolone glucuronide levels on HER+ BC. (**E**) Vanillic acid glycine levels on HER- BC. (**F**) Thyroxine levels on HER- BC. (**G**) 1-palmitoyl-2-linoleoyl-GPI (16:0/18:2) levels on HER-BC. (**H**) N-acetylphenylalanine levels on HER- BC. (**I**) Glucose-to-mannose ratio on HER- BC. Abbreviations: HER: human epidermal growth factor receptor; BC: breast cancer.
