# Supplementary material for: Causal role of blood metabolites in HER-positive and HER-negative breast cancer: a Mendelian randomization (MR) study
Source: Aging (Albany NY). 2024 Aug 2;16(15):11626–55. doi: 10.18632/aging.206042 (PMC11346783; doi:10.18632/aging.206042)
Supplement: Supplementary Tables [file aging-16-206042-s002.pdf]

SUPPLEMENTARY TABLES

Supplementary Table 1. Genetic data for the MR on the effect of epiandrosterone sulfate levels on malignant neoplasm of breast, HER-positive.

SNP	beta_X	se_X	p_X	or	or_lci95	or_uci95	F
rs10822184	−0.188421569	0.23294118	0.418583909	0.828265463	0.524669739	1.307534292	20.73820934
rs10964588	0.032182983	0.266742083	0.903966629	1.032706456	0.612239642	1.741936571	21.43573389
rs112881196	−0.435423443	0.239048617	0.068533182	0.646990646	0.404963341	1.033666145	28.35063804
rs1165191	0.257408695	0.215939321	0.23324486	1.293573696	0.847188233	1.975160704	24.91289764
rs117209214	0.127939656	0.076937053	0.096329772	1.136484421	0.977402274	1.321458803	55.18570351
rs117907084	0.003556025	0.19970231	0.985793116	1.003562355	0.678506987	1.484343449	20.06779613
rs117976748	−0.145734469	0.198434995	0.462693292	0.864387194	0.585864315	1.275321267	20.2000859
rs11932379	0.57928347	0.280559558	0.038947294	1.784759139	1.029822788	3.09311973	21.63336488
rs1335061	−0.261670892	0.227828544	0.25074445	0.769764318	0.492522646	1.203065706	20.53411176
rs140628452	0.183972838	0.217284855	0.397168127	1.201983175	0.785130387	1.840157479	20.19661709
rs148982377	0.058740377	0.020914412	0.004975624	1.060499875	1.017906564	1.104875462	1232.653533
rs149982314	0.588319122	0.501964201	0.241183397	1.800958677	0.673322603	4.817084918	20.48065325
rs17586938	0.289261488	0.244790928	0.237337653	1.335440884	0.82652201	2.157719132	21.92739899
rs17834682	0.037573221	0.316268616	0.905432532	1.038288019	0.55860467	1.929883635	21.56000559
rs1939768	0.45061658	0.236461542	0.05669363	1.569279474	0.987234142	2.494482274	34.83365566
rs34445681	−0.209590616	0.185887977	0.259526773	0.810916154	0.563306693	1.167365873	22.74255116
rs4314048	0.386768204	0.238304444	0.104589323	1.472215198	0.922831603	2.348659909	20.06542188
rs4961487	0.087461739	0.203206663	0.666899002	1.091400505	0.732843343	1.625388392	26.52156846
rs56225736	0.283035001	0.170650237	0.097202899	1.327151613	0.94986115	1.854304076	38.52168963
rs598997	0.07868796	0.224981019	0.726523497	1.081866683	0.696091029	1.681440317	21.2381214
rs76205362	0.064688883	0.17818401	0.716570982	1.066827064	0.752351399	1.512750539	20.13317837
rs9294747	−0.126756	0.220332878	0.565093039	0.880948598	0.572004488	1.356755843	26.37805868
rs949696	0.163540851	0.209913237	0.43592797	1.177673464	0.780446495	1.777078628	20.53960905
rs969114	−0.057751433	0.159153317	0.716704492	0.943884537	0.690947155	1.289415569	41.18745558
All - Inverse variance weighted	0.063053547	0.019637758	0.001323512	1.065083869	1.024867709	1.106878125	NA
All - MR Egger	0.062741091	0.023725518	0.014808268	1.06475113	1.016371646	1.115433486	NA

Abbreviations: F: F-statistic; Beta_X: genetic effect on Epiandrosterone sulfate levels; SE_X: standard error of Beta_X; p_X: *p*-value for Beta_X.

Supplementary Table 2. Genetic data for the MR on the effect of 5alpha-androstan-3beta,17beta-diol monosulfate (2) levels on malignant neoplasm of breast, HER-positive.

SNP	beta_X	se_X	p_X	or	or_lci95	or_uci95	F
rs111863675	−0.009326836	0.513639177	0.985512535	0.990716524	0.362018623	2.71123961	23.07545101
rs112881196	−0.30581922	0.167895557	0.068533182	0.736519756	0.529991393	1.023528603	48.04621488
rs11663683	−0.3421822	0.194710939	0.078852106	0.710218791	0.484898523	1.040239776	24.65996095
rs117299048	−0.331387347	0.33877409	0.327977559	0.717927028	0.36958129	1.394603115	22.85136511
rs117907084	0.003197174	0.179549622	0.985793116	1.00320229	0.705590607	1.426343867	20.91978384
rs11807828	0.277783356	0.261994272	0.28902406	1.320200152	0.789997563	2.20624534	24.84529912
rs1202220	0.293139268	0.204683138	0.152097665	1.340629484	0.897591978	2.002343446	19.88100982
rs138257623	0.101011682	0.240881413	0.674966239	1.106289566	0.68996398	1.773826808	23.80669888
rs139943078	0.016669697	0.486205337	0.972649645	1.016809412	0.392078673	2.636974289	20.93272933

rs141113556	0.207232641	0.552168523	0.707432256	1.230268749	0.416854657	3.630908692	20.33773588
rs143358204	−0.075303679	0.268048007	0.778761366	0.927461793	0.548439793	1.568422622	19.66687909
rs146308324	−0.502091443	0.253103617	0.047284976	0.605263461	0.368551606	0.99400966	19.67591162
rs146802218	0.12393409	0.211983744	0.55879008	1.131941262	0.747101632	1.71501569	22.04457795
rs147344701	−0.129526678	0.225266144	0.565295364	0.878511151	0.564932897	1.366147814	21.38021978
rs148982377	0.071205907	0.025352743	0.004975624	1.073802307	1.021747638	1.128508989	424.9030325
rs150754448	0.132156646	0.214030393	0.536926923	1.141287083	0.750254404	1.736126038	21.45243931
rs2123886	0.15836279	0.154357665	0.304916548	1.171591159	0.865733431	1.58550634	36.55078129
rs342165	0.146255663	0.192725862	0.447924777	1.157492078	0.793352987	1.688766453	20.43767199
rs35192168	−0.060529064	0.201004385	0.763313036	0.941266411	0.634766758	1.395760642	21.44885522
rs4149056	0.422960835	0.173259937	0.014638855	1.52647451	1.086945381	2.143736449	27.01568196
rs548244437	0.542581896	0.29577842	0.066591649	1.720443138	0.963537563	3.071934823	24.45539684
rs56225736	0.291948112	0.176024217	0.097202899	1.339033536	0.948323725	1.890715968	32.08251119
rs6807359	0.086811931	0.231770218	0.707987476	1.090691535	0.692492621	1.717863829	20.08732606
rs6843105	0.107429998	0.185915153	0.563369293	1.113412917	0.773396286	1.602914762	23.44067997
rs7139537	0.099068951	0.185554757	0.593406002	1.104142428	0.767498792	1.588446152	25.17973585
rs75121365	0.120666996	0.126305891	0.339398079	1.128249138	0.880828311	1.445169394	20.73057531
rs772736	−0.152966163	0.383980642	0.690357862	0.858158758	0.404312081	1.82145548	21.26363088
rs78424818	0.28440662	0.211148087	0.177995262	1.328973207	0.878584113	2.010245531	20.18660501
rs79728496	−0.202806567	0.220141111	0.356916198	0.816436151	0.530315512	1.256927196	19.94569702
rs9422240	0.20860023	0.218748032	0.340281279	1.231952403	0.802401708	1.891454998	19.96396173
rs9933711	0.119312043	0.213005107	0.57538599	1.126721449	0.742169247	1.710527928	22.56486913
All - Inverse variance weighted	0.07112927	0.021948953	0.0011925	1.073720017	1.028508118	1.120919373	NA
All - MR Egger	0.060598849	0.028310409	0.040851566	1.062472617	1.005123527	1.123093859	NA

Abbreviations: F: F-statistic; Beta_X: genetic effect on 5alpha-androstan-3beta,17beta-diol monosulfate (2) levels; SE_X: standard error of Beta_X; p_X: *p*-value for Beta_X.

Supplementary Table 3. Genetic data for the MR on the effect of glycohyocholate levels on malignant neoplasm of breast, HER-positive.

SNP	beta_X	se_X	p_X	or	or_lci95	or_uci95	F
rs11590130	0.016348	0.201423	0.935312	1.016483	0.684928	1.508534	20.20511
rs12455551	−0.09816	0.199881	0.623366	0.906505	0.612671	1.341259	19.98324
rs144012054	−0.33053	0.254222	0.193543	0.718542	0.43657	1.182634	22.42442
rs144846334	−0.23794	0.243737	0.328958	0.788251	0.488868	1.270976	27.61669
rs281377	−0.23615	0.187632	0.208176	0.78966	0.546669	1.140658	24.39526
rs3110095	−0.2343	0.139719	0.093561	0.791128	0.60161	1.040346	43.51957
rs3802548	−0.01367	0.158	0.931068	0.986426	0.723723	1.344487	31.89631
rs495360	−0.02041	0.173046	0.906093	0.979793	0.697966	1.375417	27.15487
rs55971546	−0.31575	0.159354	0.04754	0.72924	0.533612	0.996586	37.17743
rs6135632	0.05405	0.182769	0.767437	1.055537	0.737731	1.510252	20.60889
rs62471957	−0.29196	0.208418	0.161258	0.746796	0.496356	1.123598	26.20432
rs62510166	−0.31292	0.212843	0.141511	0.731309	0.481864	1.109882	20.35706
rs6913415	−0.08699	0.197944	0.660333	0.916689	0.621912	1.351186	20.66338
rs74377562	−0.32945	0.174815	0.059487	0.719318	0.51064	1.013273	25.87804
rs79430699	−0.00756	0.267961	0.977496	0.99247	0.586981	1.678072	20.31629
rs80129176	−0.17817	0.285062	0.531954	0.836799	0.478599	1.46309	22.44105

All - Inverse variance weighted	−0.16328	0.048004	0.000671	0.849354	0.773084	0.933148	NA
All - MR Egger	−0.30179	0.105753	0.012756	0.739496	0.601059	0.909817	NA

Abbreviations: F: F-statistic; Beta_X: genetic effect on Glycohyocholate levels; SE_X: standard error of Beta_X; p_X: *p*-value for Beta_X

Supplementary Table 4. Genetic data for the MR on the effect of etiocholanolone glucuronide levels on malignant neoplasm of breast, HER-positive.

SNP	beta_X	se_X	p_X	or	or_lci95	or_uci95	F
rs111975045	0.095969	0.181569	0.597115	1.100725	0.771123	1.571209	20.9241
rs112114309	−0.05323	0.191212	0.780736	0.948166	0.65181	1.379264	20.02932
rs113018018	0.343348	0.204587	0.093299	1.409659	0.943986	2.105051	22.52342
rs117736840	0.282134	0.258356	0.274818	1.325956	0.79912	2.200119	22.65326
rs138529890	0.339581	0.169448	0.045065	1.404359	1.007491	1.957561	50.75351
rs141232858	0.091693	0.050264	0.068119	1.096028	0.993198	1.209504	750.6996
rs141909149	0.48145	0.356618	0.177002	1.618419	0.80451	3.255746	20.04527
rs148982377	0.255139	0.090842	0.004976	1.290641	1.080138	1.542168	49.29313
rs17339782	-0.48544	0.194454	0.012546	0.615428	0.420392	0.900949	19.7898
rs231620	0.047124	0.222408	0.832199	1.048252	0.677873	1.621001	19.54917
rs28360521	0.10378	0.269853	0.700549	1.109356	0.653684	1.882672	21.11227
rs3857868	0.271677	0.178246	0.127466	1.312163	0.925256	1.86086	32.03785
rs4694077	-0.06806	0.21386	0.750293	0.934203	0.614327	1.420635	20.24474
rs6698394	0.070593	0.211988	0.739129	1.073145	0.708289	1.625945	22.94598
rs72794638	−0.06825	0.19026	0.719797	0.934025	0.643289	1.35616	59.20234
rs72941955	0.249276	0.356158	0.483989	1.283096	0.638397	2.578857	20.43926
rs75936454	0.037777	0.183449	0.836848	1.0385	0.724856	1.487856	20.21996
rs78320625	0.28462	0.200691	0.156133	1.329257	0.896968	1.969884	19.72805
rs8042104	0.154115	0.214331	0.47211	1.166625	0.766459	1.775718	21.61875
rs9957928	−0.06675	0.206792	0.746847	0.935427	0.623713	1.402926	24.8461
All - Inverse variance weighted	0.113156	0.035173	0.001295	1.119807	1.04521	1.199728	NA
All - MR Egger	0.112137	0.053207	0.049354	1.118666	1.007882	1.241627	NA

Abbreviations: F: F-statistic; Beta_X: genetic effect on Etiocholanolone glucuronide levels; SE_X: standard error of Beta_X; p_X: *p*-value for Beta_X.

Supplementary Table 5. Genetic data for the MR on the effect of vanillic acid glycine levels on malignant neoplasm of breast, HER-negative.

SNP	beta_X	se_X	p_X	or	or_lci95	or_uci95	F
rs1047891	0.135427	0.241475	0.574912	1.145026	0.713292	1.838074	23.2309
rs10845632	0.18359	0.228901	0.422525	1.201523	0.767163	1.881815	24.04263
rs114538296	−0.0399	0.176104	0.82074	0.960882	0.680404	1.356978	43.54908
rs117841983	−0.08292	0.13728	0.545846	0.920428	0.703291	1.204605	20.53855
rs150827829	0.263888	0.205362	0.198797	1.301982	0.870557	1.94721	20.18708
rs187352208	0.195583	0.056102	0.00049	1.21602	1.089396	1.357363	21.58818
rs34199055	0.593037	0.225676	0.008593	1.809476	1.162662	2.816127	29.9908
rs3799340	−0.18685	0.17779	0.29328	0.829569	0.585484	1.175413	47.17725
rs395438	0.167138	0.294723	0.570646	1.181917	0.663305	2.106011	21.08149

rs4043197	−0.12212	0.324078	0.706305	0.885042	0.468924	1.670417	20.64513
rs4355631	−0.15312	0.254099	0.546778	0.858028	0.521444	1.41187	20.46171
rs55991483	−0.18764	0.257874	0.466845	0.828917	0.500039	1.3741	20.2511
rs7143814	0.416955	0.25529	0.102414	1.517334	0.919971	2.502581	21.81484
rs72817365	0.153579	0.205552	0.454969	1.166	0.779345	1.744487	20.86364
rs73233368	−0.1427	0.266131	0.591816	0.867013	0.514624	1.460701	20.93719
rs73432460	0.308528	0.269	0.251405	1.361419	0.803553	2.306585	20.47054
rs74970545	0.133812	0.359918	0.710053	1.143178	0.564606	2.314634	25.15526
rs7664352	0.023403	0.277305	0.932743	1.023679	0.594452	1.762831	20.71519
rs78850110	0.068632	0.217174	0.751983	1.071042	0.699753	1.639338	22.50003
rs7915981	−0.11412	0.262514	0.663762	0.89215	0.533312	1.492431	20.14542
rs8106748	0.156164	0.147007	0.288102	1.169018	0.876368	1.559395	59.55688
rs9300713	0.263685	0.249369	0.290325	1.301718	0.798454	2.122187	23.38335
rs9835855	0.293402	0.218799	0.179932	1.340981	0.873327	2.059057	21.18513
All - Inverse variance weighted	0.130564	0.035981	0.000285	1.139471	1.061881	1.22273	NA
All - MR Egger	0.161645	0.052462	0.005663	1.175443	1.060585	1.30274	NA

Abbreviations: F: F-statistic; Beta_X: genetic effect on Vanillic acid glycine levels; SE_X: standard error of Beta_X; p_X: *p*-value for Beta_X.

Supplementary Table 6. Genetic data for the MR on the effect of thyroxine levels on malignant neoplasm of breast, HER-negative.

SNP	beta_X	se_X	p_X	or	or_lci95	or_uci95	F
rs10745656	0.132547	0.317067	0.675917	1.141733	0.613298	2.125481	19.99626
rs111892516	0.129683	0.444183	0.770319	1.138467	0.476677	2.719045	20.35275
rs114685250	1.125	0.331035	0.000678	3.080218	1.6099	5.893373	21.67381
rs1169288	−0.35188	0.221182	0.111631	0.703365	0.455939	1.085062	29.97077
rs117341978	0.37451	0.33825	0.268207	1.454279	0.749417	2.822095	19.96895
rs117672019	0.394313	0.9104	0.664927	1.483365	0.249058	8.834787	20.47427
rs11779237	0.352126	0.286673	0.219326	1.422088	0.810785	2.494291	23.69207
rs13094078	0.223959	0.183097	0.221264	1.25102	0.873794	1.791098	20.30568
rs144476500	−0.13466	0.267005	0.614019	0.874011	0.51789	1.475014	26.22235
rs146643391	0.216047	0.25445	0.395839	1.241161	0.753765	2.043713	20.16352
rs16844401	0.434456	0.287088	0.130198	1.544122	0.879646	2.710538	21.95951
rs17361586	0.4097	0.359475	0.254404	1.506366	0.744628	3.047344	22.47474
rs56357032	0.425139	0.317509	0.180576	1.529803	0.821045	2.850391	21.10301
rs58541168	0.28832	0.180453	0.110096	1.334184	0.936723	1.900292	20.47729
rs6078009	0.560027	0.300191	0.062101	1.750721	0.972052	3.153147	23.04986
rs6722076	0.102134	0.190838	0.592519	1.107532	0.761925	1.609907	38.40639
rs72869950	0.542499	0.514439	0.291634	1.7203	0.627632	4.715236	20.67701
rs73087204	0.42326	0.226859	0.062078	1.526931	0.978842	2.381913	21.17641
rs74454704	0.082849	0.279324	0.766767	1.086378	0.62837	1.878222	21.44002
rs78013831	0.428069	0.202577	0.034591	1.534291	1.031504	2.282152	23.51178
rs79132259	−0.36707	0.296642	0.215937	0.692764	0.387328	1.239058	19.56237
All - Inverse variance weighted	0.233614	0.066072	0.000407	1.263156	1.109726	1.4378	NA
All - MR Egger	0.408993	0.16511	0.022814	1.505302	1.089129	2.080501	NA

Abbreviations: F: F-statistic; Beta_X: genetic effect on Thyroxine levels; SE_X: standard error of Beta_X; p_X: *p*-value for Beta_X.

Supplementary Table 7. Genetic data for the MR on the effect of 1-palmitoyl-2-linoleoyl-GPI (16:0/18:2) levels on malignant neoplasm of breast, HER-negative.

SNP	beta_X	se_X	p_X	or	or_lci95	or_uci95	F
rs10197694	0.109425	0.267058	0.681994	1.115637	0.660995	1.882988	21.69864
rs10468017	−0.00197	0.257232	0.993877	0.998028	0.602813	1.652353	21.12909
rs113703749	0.024403	0.355626	0.945293	1.024703	0.510367	2.057374	20.6024
rs141418237	−0.32441	0.255004	0.203308	0.722952	0.438577	1.191718	20.0654
rs144800978	0.397625	0.5747	0.489011	1.488286	0.482494	4.59072	20.02776
rs17119241	0.021194	0.266099	0.936518	1.02142	0.606312	1.72073	22.05837
rs174564	−0.14958	0.105179	0.154987	0.861071	0.700663	1.058203	128.2794
rs192787921	−0.04159	0.143314	0.771667	0.959264	0.724348	1.270367	22.96185
rs2045700	0.009159	0.265864	0.972519	1.009201	0.599335	1.699362	21.36032
rs2424708	−0.29237	0.202258	0.14831	0.746493	0.502181	1.109663	36.53821
rs2701180	−0.4721	0.265588	0.075475	0.62369	0.370592	1.049645	21.15642
rs390035	−0.27102	0.26163	0.300255	0.762602	0.456662	1.273509	21.8915
rs554067	−0.28897	0.257274	0.261355	0.749036	0.452383	1.240219	22.33709
rs56043834	−0.37799	0.264855	0.153532	0.685236	0.407748	1.151567	19.55945
rs6133675	−0.30561	0.290871	0.29341	0.736674	0.416563	1.302776	21.32047
rs641971	−0.24098	0.266641	0.366123	0.785858	0.465988	1.325297	19.77715
rs6491411	−0.25384	0.280509	0.365514	0.77582	0.447699	1.34442	21.33727
rs7412	−0.07137	0.270175	0.791666	0.931121	0.548313	1.581188	31.0331
rs75917318	−0.39402	0.276574	0.154265	0.674344	0.392154	1.159595	21.64591
rs78519165	0.07761	0.246901	0.753266	1.080701	0.6661	1.753362	20.92825
rs8736	−0.12695	0.097302	0.191987	0.880775	0.727846	1.065837	161.469
All - Inverse variance weighted	−0.14864	0.045047	0.000968	0.861875	0.78904	0.941433	NA
All - MR Egger	−0.05745	0.100287	0.573469	0.94417	0.775682	1.149256	NA

Abbreviations: F: F-statistic; Beta_X: genetic effect on 1-palmitoyl-2-linoleoyl-GPI (16:0/18:2) levels; SE_X: standard error of Beta_X; p_X: *p*-value for Beta_X.

Supplementary Table 8. Genetic data for the MR on the effect of N-acetylphenylalanine levels on malignant neoplasm of breast, HER-negative.

SNP	beta_X	se_X	p_X	or	or_lci95	or_uci95	F
rs10436897	0.162257	0.371858	0.662588	1.176163	0.567461	2.437806	19.53623
rs111528892	0.273874	0.417734	0.512069	1.31505	0.579909	2.982116	20.47289
rs116075297	0.154515	0.169156	0.361007	1.167092	0.837754	1.625901	19.79232
rs117013634	0.172937	0.365399	0.636012	1.188791	0.58086	2.432984	19.79306
rs12444803	0.212904	0.272664	0.434903	1.237266	0.725048	2.111346	21.77973
rs138252727	−0.09767	0.221159	0.658771	0.906951	0.587935	1.399068	19.50911
rs139658164	−0.22814	0.427795	0.593832	0.796013	0.34417	1.841056	23.5126
rs149251158	−0.17558	0.17629	0.319264	0.83897	0.593862	1.185244	36.48982
rs17707159	0.025432	0.291646	0.93051	1.025758	0.57915	1.816768	20.4308
rs2010501	0.039769	0.272372	0.883914	1.04057	0.610132	1.774676	19.66764
rs2149614	0.329545	0.280591	0.240207	1.390336	0.802188	2.409702	20.63435
rs2328895	0.082115	0.133183	0.537526	1.085581	0.83617	1.409386	98.4315
rs2360636	0.508759	0.284186	0.073416	1.663226	0.9529	2.903052	21.86603
rs3763785	0.358775	0.266672	0.178502	1.431575	0.848826	2.414402	20.44586

rs61757081	0.426654	0.320268	0.182803	1.532122	0.817853	2.870193	22.4386
rs62576887	−0.17678	0.329819	0.59197	0.837966	0.439015	1.599463	20.03196
rs6868892	0.44287	0.288824	0.125188	1.557171	0.884064	2.742765	20.16622
rs7108760	0.041507	0.115648	0.719663	1.042381	0.830968	1.30758	179.082
rs72870683	0.114036	0.266764	0.669031	1.120792	0.664433	1.890599	25.41432
rs7604588	0.128698	0.044702	0.00399	1.137346	1.041937	1.241493	926.7864
rs76260331	−0.04938	0.329861	0.881006	0.951821	0.498623	1.816931	19.96712
rs79031621	0.033579	0.252513	0.894209	1.03415	0.630434	1.696395	20.27854
rs9852875	−0.02209	0.443771	0.960295	0.97815	0.409883	2.334268	21.30936
rs9957535	0.042629	0.344942	0.901645	1.043551	0.530754	2.051795	21.09939
All - Inverse variance weighted	0.112196	0.033219	0.000731	1.118733	1.048214	1.193995	NA
All - MR Egger	0.070678	0.052114	0.18879	1.073235	0.969024	1.188655	NA

Abbreviations: F: F-statistic; Beta_X: genetic effect on N-acetylphenylalanine levels; SE_X: standard error of Beta_X; p_X: *p*-value for Beta_X.

Supplementary Table 9. Genetic data for the MR on the effect of glucose-to-mannose ratio on malignant neoplasm of breast, HER-negative.

SNP	beta_X	se_X	p_X	or	or_lci95	or_uci95	F
rs10487783	0.184665	0.313782	0.556187	1.202816	0.650283	2.224824	19.95859
rs11076008	0.004923	0.305119	0.987127	1.004935	0.552605	1.827515	19.71775
rs11147164	−0.02491	0.185323	0.89307	0.975396	0.678314	1.402593	21.8003
rs11183167	0.192068	0.239798	0.423157	1.211753	0.757345	1.938806	21.86084
rs1260326	0.162462	0.070699	0.021566	1.176403	1.024179	1.351254	335.8678
rs1260815	0.027013	0.300905	0.928469	1.027381	0.569634	1.852964	19.59771
rs13151496	0.260273	0.211747	0.219007	1.297285	0.856629	1.964616	21.39685
rs140698139	0.204595	0.270158	0.44886	1.227028	0.722589	2.083616	19.95859
rs141341042	0.121498	0.20112	0.545774	1.129187	0.761323	1.674799	20.49849
rs1434218	0.117687	0.277163	0.671119	1.124892	0.653409	1.936585	20.55981
rs144226876	0.170973	0.309431	0.580578	1.186459	0.646933	2.175934	20.58319
rs144897897	−0.04263	0.249589	0.864376	0.958265	0.587532	1.56293	20.20088
rs146434711	0.094234	0.22554	0.676081	1.098817	0.706223	1.709656	19.63824
rs1868856	0.241477	0.265734	0.363499	1.273128	0.756267	2.143232	20.31357
rs2897514	−0.08285	0.281854	0.768795	0.920488	0.529785	1.599325	20.02076
rs407109	−0.13179	0.298444	0.658786	0.876525	0.488342	1.573275	22.50079
rs4143117	0.190045	0.255668	0.457284	1.209304	0.732666	1.996019	19.71288
rs56243479	0.224412	0.225723	0.32013	1.251586	0.804121	1.94805	21.52789
rs60314390	−0.03039	0.234005	0.896663	0.970065	0.613213	1.534583	20.90927
rs73430632	0.175201	0.303778	0.564114	1.191486	0.656914	2.161073	20.27928
rs74833360	0.185068	0.274262	0.499813	1.2033	0.702938	2.059827	23.65132
rs75139539	0.046835	0.226002	0.83583	1.047949	0.672919	1.631989	21.07131
rs7818895	0.434803	0.275897	0.115035	1.544659	0.899465	2.652654	20.06782
rs79405811	0.189572	0.207939	0.361941	1.208733	0.804135	1.816902	21.77785
rs8049404	0.308263	0.265143	0.244981	1.361058	0.809435	2.288608	22.3594
rs977895	0.108016	0.296469	0.715602	1.114066	0.623091	1.991914	20.22298

All - Inverse variance weighted	0.137918	0.040991	0.000767	1.147881	1.059266	1.24391	NA
All - MR Egger	0.132366	0.080175	0.111774	1.141526	0.975527	1.335773	NA

Abbreviations: F: F-statistic; Beta_X: genetic effect on Glucose-to-mannose ratio; SE_X: standard error of Beta_X; p_X: *p*-value for Beta_X.

Supplementary Table 10. Genetic data for the MR on the effect of gamma-glutamyl glutamate levels on malignant neoplasm of breast, HER-positive.

SNP	beta_X	se_X	p_X	or	or_lci95	or_uci95	F
rs10918906	0.224046	0.210365	0.286859	1.251129	0.828392	1.889591	23.65602
rs10948077	0.109634	0.199475	0.582584	1.11587	0.754774	1.64972	41.30163
rs113421406	−0.16811	0.442487	0.703996	0.845257	0.355088	2.012061	19.82452
rs113558190	0.01412	0.260835	0.95683	1.01422	0.608282	1.691061	20.01361
rs113567875	1.18976	0.474075	0.012085	3.286294	1.297674	8.322374	20.41966
rs113874211	−0.75929	0.414896	0.06724	0.467999	0.207529	1.055388	21.30396
rs117838486	0.457242	0.354576	0.197208	1.579712	0.788418	3.165184	19.57548
rs12628903	1.549331	0.696083	0.026029	4.708318	1.203229	18.42398	21.02661
rs1324191	0.376135	0.304447	0.216656	1.456644	0.802053	2.645476	19.93714
rs13385401	0.077626	0.290218	0.789103	1.080719	0.611891	1.908759	23.87765
rs1410284	0.230933	0.212766	0.277752	1.259775	0.8302	1.911628	27.36363
rs142947473	−0.00357	0.267129	0.989333	0.996435	0.590289	1.682029	21.23784
rs1479402	−0.03973	0.404449	0.92174	0.961045	0.434981	2.12333	19.67343
rs1702339	−0.41855	0.437569	0.338807	0.658003	0.279102	1.551294	20.34053
rs17729572	0.22393	0.331842	0.499797	1.250984	0.652803	2.397294	20.49173
rs1937855	−0.0792	0.261496	0.761977	0.923852	0.553367	1.542382	22.24685
rs2209169	0.301682	0.278392	0.278516	1.352132	0.783514	2.333411	20.23599
rs34618040	0.194237	0.237317	0.413087	1.214384	0.76269	1.93359	20.76366
rs3859862	0.045667	0.185696	0.805742	1.046726	0.727387	1.506261	46.12258
rs4781721	0.637161	0.240791	0.008142	1.891104	1.179641	3.031662	25.23359
rs541090833	0.282286	0.32908	0.391	1.326158	0.695788	2.52763	19.67458
rs72768751	0.617449	0.382692	0.10665	1.854193	0.875791	3.925628	21.30536
rs74622465	−0.61806	0.327021	0.05876	0.538987	0.283931	1.023161	21.42821
rs7614103	−0.0383	0.272212	0.888105	0.962423	0.564488	1.640882	21.59485
rs76502482	−0.04564	0.407468	0.910824	0.95539	0.42987	2.123363	24.79271
All - Inverse variance weighted	0.142585	0.066054	0.030881	1.153251	1.013205	1.312655	NA
All - MR Egger	0.014249	0.181106	0.937971	1.014351	0.711259	1.4466	NA

Abbreviations: F: F-statistic; Beta_X: genetic effect on Gamma-glutamyl glutamate levels; SE_X: standard error of Beta_X; p_X: *p*-value for Beta_X.

Supplementary Table 11. Genetic data for the MR on the effect of X-12849 levels on malignant neoplasm of breast, HER-positive.

SNP	beta_X	se_X	p_X	or	or_lci95	or_uci95	F
rs111295425	−0.06402	0.182714	0.72607	0.93799	0.655645	1.341925	20.29968
rs11487435	0.067219	0.272789	0.805362	1.06953	0.6266	1.825556	22.24892
rs12872612	−0.2033	0.274097	0.458272	0.816036	0.476862	1.396451	20.78645
rs13386620	0.249797	0.255629	0.328479	1.283764	0.777838	2.118758	22.27942

rs150860204	−0.59147	0.445726	0.184516	0.553514	0.231057	1.325982	21.76679
rs17091434	0.176696	0.238975	0.459668	1.193268	0.746997	1.906152	21.36393
rs186129911	0.017042	0.138513	0.902078	1.017188	0.775347	1.334463	22.59548
rs1876254	0.234897	0.257684	0.361995	1.264778	0.763255	2.095845	20.97361
rs2206890	−0.3582	0.295554	0.225522	0.698931	0.39161	1.247425	21.18312
rs2833587	−0.04387	0.23544	0.852179	0.957077	0.603304	1.5183	24.97789
rs2916610	−0.60233	0.228838	0.008485	0.547533	0.349639	0.857435	20.17495
rs2942211	−0.05053	0.256245	0.843681	0.950727	0.575355	1.570999	19.55932
rs35326271	0.11037	0.262913	0.674635	1.116691	0.667018	1.869513	21.37654
rs3828210	−0.00436	0.289035	0.987953	0.995645	0.565032	1.754432	20.36693
rs62321483	−0.06282	0.24128	0.79459	0.939114	0.585244	1.506953	19.66226
rs6716856	−0.22686	0.296503	0.444199	0.797031	0.445745	1.425161	21.91351
rs72611556	−0.13348	0.132526	0.313833	0.875044	0.674872	1.134587	21.28985
rs73115392	−0.54438	0.265697	0.040473	0.5802	0.344677	0.97666	21.38965
rs76447666	−0.3912	0.526486	0.457459	0.676246	0.240963	1.897834	20.85577
rs76738915	−0.09551	0.386448	0.804789	0.908907	0.426156	1.938521	20.90233
rs79918116	−0.6315	0.341097	0.064113	0.531792	0.272518	1.037741	20.14291
rs9531784	−0.39232	0.26643	0.140889	0.675491	0.40071	1.1387	20.4065
All - Inverse variance weighted	−0.11167	0.05099	0.028522	0.89434	0.809281	0.988339	NA
All - MR Egger	−0.1982	0.103165	0.06907	0.820203	0.670047	1.004007	NA

Abbreviations: F: F-statistic; Beta_X: genetic effect on X-12849 levels; SE_X: standard error of Beta_X; p_X: *p*-value for Beta_X.

Supplementary Table 12. Genetic data for the MR on the effect of gamma-glutamyl glutamate levels on malignant neoplasm of breast, HER-negative.

SNP	beta_X	se_X	p_X	or	or_lci95	or_uci95	F
rs10918906	0.101604	0.167793	0.544828	1.106944	0.796705	1.537992	23.65602
rs10948077	0.246031	0.159203	0.122253	1.278939	0.936124	1.747296	41.30163
rs113421406	−0.25568	0.356973	0.473842	0.77439	0.384679	1.558913	19.82452
rs113558190	−0.13508	0.207103	0.514245	0.873645	0.582164	1.311066	20.01361
rs113567875	0.048458	0.382438	0.899172	1.049651	0.496029	2.221174	20.41966
rs113874211	−0.1038	0.336623	0.757813	0.901406	0.465995	1.743652	21.30396
rs117838486	0.21209	0.284623	0.456174	1.236259	0.707675	2.159658	19.57548
rs12628903	−0.52952	0.569359	0.352353	0.588886	0.192922	1.797542	21.02661
rs1324191	0.367854	0.243098	0.130231	1.444632	0.897073	2.32641	19.93714
rs13385401	−0.07421	0.232106	0.749172	0.928475	0.589112	1.463331	23.87765
rs1410284	0.240185	0.16971	0.15699	1.271484	0.911698	1.773254	27.36363
rs142947473	−0.03101	0.213287	0.884417	0.96947	0.638235	1.47261	21.23784
rs1479402	−0.53154	0.327986	0.105101	0.5877	0.309007	1.117746	19.67343
rs1702339	−0.00303	0.349937	0.993084	0.996971	0.502123	1.979499	20.34053
rs17729572	−0.16542	0.264892	0.532323	0.847541	0.504289	1.424432	20.49173
rs1937855	0.449422	0.208256	0.030925	1.567406	1.042103	2.357504	22.24685
rs2209169	−0.02698	0.222324	0.903418	0.973383	0.62956	1.504977	20.23599
rs34618040	0.039344	0.189479	0.83551	1.040128	0.717462	1.507907	20.76366
rs3859862	0.080187	0.148064	0.588116	1.08349	0.810569	1.448304	46.12258
rs4781721	0.260756	0.19233	0.175171	1.29791	0.890287	1.892166	25.23359
rs541090833	0.236721	0.264961	0.371635	1.267087	0.753819	2.129835	19.67458

rs72768751	−0.07131	0.304854	0.815062	0.931177	0.512313	1.692501	21.30536
rs74622465	0.192225	0.259384	0.458642	1.211944	0.728938	2.014997	21.42821
rs7614103	0.222893	0.217264	0.304935	1.249687	0.816324	1.913111	21.59485
rs76502482	−0.17329	0.324639	0.593476	0.84089	0.445042	1.588832	24.79271
All - Inverse variance weighted	0.092333	0.045861	0.044082	1.09673	1.002448	1.199879	NA
All - MR Egger	−0.15293	0.125031	0.233654	0.858187	0.671666	1.096505	NA

Abbreviations: F: F-statistic; Beta_X: genetic effect on Gamma-glutamyl glutamate levels; SE_X: standard error of Beta_X; p_X: *p*-value for Beta_X.

Supplementary Table 13. Genetic data for the MR on the effect of X-12849 levels on malignant neoplasm of breast, HER-negative.

	beta_X	se_X	p_X	or	or_lci95	or_uci95	F
rs111295425	−0.12127	0.145564	0.404772	0.885792	0.665925	1.178251	20.29968
rs11487435	−0.27573	0.218024	0.205987	0.759018	0.49507	1.163691	22.24892
rs12872612	−0.08852	0.217982	0.684689	0.915288	0.597045	1.403163	20.78645
rs13386620	0.076545	0.203647	0.707012	1.079551	0.724261	1.609131	22.27942
rs150860204	−0.40261	0.362336	0.266509	0.668576	0.328643	1.36012	21.76679
rs17091434	0.03062	0.190521	0.872317	1.031094	0.709779	1.497866	21.36393
rs186129911	−0.10676	0.109875	0.331241	0.898745	0.724618	1.114714	22.59548
rs1876254	0.300179	0.205333	0.143765	1.350101	0.902783	2.01906	20.97361
rs2206890	0.022434	0.23593	0.924247	1.022687	0.644044	1.623941	21.18312
rs2833587	−0.12703	0.187742	0.498658	0.88071	0.60957	1.272455	24.97789
rs2916610	−0.02975	0.179851	0.868629	0.970691	0.682321	1.380934	20.17495
rs2942211	0.141589	0.204329	0.488343	1.152103	0.771904	1.719569	19.55932
rs35326271	−0.00962	0.209668	0.963397	0.990424	0.656672	1.493806	21.37654
rs3828210	−0.46252	0.231383	0.045617	0.629697	0.400105	0.991034	20.36693
rs62321483	0.028356	0.192333	0.882793	1.028761	0.705663	1.499796	19.66226
rs6716856	−0.44285	0.235426	0.059964	0.642204	0.404832	1.018759	21.91351
rs72611556	−0.11802	0.105711	0.264224	0.888676	0.722372	1.093268	21.28985
rs73115392	−0.10132	0.21201	0.632719	0.903644	0.596391	1.369189	21.38965
rs76447666	0.591765	0.425366	0.164168	1.807175	0.785093	4.159867	20.85577
rs76738915	0.027982	0.30992	0.928059	1.028377	0.5602	1.887823	20.90233
rs79918116	−0.78018	0.284595	0.006118	0.458323	0.262373	0.800616	20.14291
rs9531784	0.047001	0.212845	0.82523	1.048123	0.690614	1.590703	20.4065
All - Inverse variance weighted	−0.0829	0.041897	0.047855	0.920443	0.847877	0.999219	NA
All - MR Egger	−0.163	0.084382	0.067685	0.849587	0.720079	1.002387	NA

Abbreviations: F: F-statistic; Beta_X: genetic effect on X-12849 levels; SE_X: standard error of Beta_X; p_X: *p*-value for Beta_X.

Supplementary Table 14. Sensitivity analyses of metabolites on malignant neoplasm of breast, HER-positive.

Exposure	Outcome	Number of IVs	Method	Heterogeneity test		MR-Egger pleiotropy test	MR-PRESSO global outlier test			
				Q	*p*-value	Intercept	*p*-value	RSSobs	*p*-value	Outlier
Epiandrosterone sulfate levels	Malignant neoplasm of breast, HER-negative	24	MR Egger	25.87971	0.256882	0.000152	0.9805	27.17973	0.487	None
			IVW	25.88043	0.306543					
5alpha-androstan-	Malignant neoplasm of	31	MR Egger	31.6985	0.333262	0.00356	0.553914	32.80531	0.52	None

3beta,17beta-diol monosulfate (2) levels	breast, HER-negative		IVW	32.09051	0.36333					
Glycohyocholate levels	Malignant neoplasm of breast, HER-negative	16	MR Egger	5.852484	0.970074	0.018193	0.163704	9.274069	0.919	None
			IVW	8.013065	0.923258					
Etiocholanolone glucuronide levels	Malignant neoplasm of breast, HER-negative	20	MR Egger	21.79846	0.241056	0.000248	0.979458	24.06592	0.347	None
			IVW	21.79928	0.294359					

Supplementary Table 15. Sensitivity analyses of metabolites on malignant neoplasm of breast, HER-negative.

Exposure	Outcome	Number of IVs	Method	Heterogeneity test		MR-Egger pleiotropy test		MR-PRESSO global outlier test		
				Q	*p*-value	Intercept	*p*-value	RSSobs	*p*-value	Outlier
Vanillic acid glycine levels	Malignant neoplasm of breast, HER-negative	23	MR Egger	20.02221	0.519855	−0.00709	0.42472	24.52811	0.521	None
			IVW	20.68498	0.540273					
Thyroxine levels	Malignant neoplasm of breast, HER-negative	21	MR Egger	24.20825	0.188285	−0.02834	0.261528	28.62793	0.215	None
			IVW	25.91447	0.168655					
1-palmitoyl-2-linoleoyl-GPI (16:0/18:2) levels	Malignant neoplasm of breast, HER-negative	21	MR Egger	8.720561	0.977838	−0.0128	0.321558	10.44594	0.982	None
			IVW	9.756483	0.972376					
N-acetylphenylalanine levels	Malignant neoplasm of breast, HER-negative	24	MR Egger	11.15759	0.972428	0.010031	0.31239	13.29427	0.973	None
			IVW	12.22665	0.966824					
Glucose-to-mannose ratio	Malignant neoplasm of breast, HER-negative	26	MR Egger	6.401489	0.99987	0.000908	0.936455	6.924869	1	None
			IVW	6.40798	0.999935					

Supplementary Table 16. Sensitivity analyses of metabolites on malignant neoplasm of breast, HER-positive/negative.

Exposure	Outcome	Number of IVs	Method	Heterogeneity test		MR-Egger pleiotropy test		MR-PRESSO global outlier test		
				Q	*p*-value	Intercept	*p*-value	RSSobs	*p*-value	Outlier
Gamma-glutamyl glutamate levels	Malignant neoplasm of breast, HER-positive	25	MR Egger	13.74344	0.933983	0.028479	0.046076	19.61343	0.83	None
			IVW	18.18968	0.793711					
X-12849 levels	Malignant neoplasm of breast, HER-positive	22	MR Egger	21.04402	0.394546	0.012455	0.287808	23.64717	0.447	None
			IVW	22.29876	0.382472					
Gamma-glutamyl glutamate levels	Malignant neoplasm of breast, HER-negative	25	MR Egger	30.99398	0.122941	0.014923	0.453749	33.63561	0.166	None
			IVW	31.77663	0.132629					
X-12849 levels	Malignant neoplasm of breast, HER-negative	22	MR Egger	19.83662	0.468191	0.013453	0.34612	0.514	22.64835	None
			IVW	20.76764	0.473211					
